# Diversity and distribution of mitochondrial DNA in non-Austronesian-speaking Taiwanese individuals

**DOI:** 10.1038/s41439-022-00228-3

**Published:** 2023-01-18

**Authors:** Marie Lin, Jean A. Trejaut

**Affiliations:** Molecular Anthropology and Transfusion Medicine Research Laboratory, Mackay Memorial Hospital, Taipei, Taiwan

**Keywords:** Structural variation, Molecular biology

## Abstract

Many studies have described the diversity of Austronesian-speaking Taiwanese people to shed more light on their origin and their connection with the “Out of Taiwan” migrations. However, the genetic relationship between the non-Austronesian-speaking groups of Taiwan and the populations of continental Asia is still unclear. Here, we studied the diversity of mtDNA in 767 non-Austronesian speakers from 16 locations in Taiwan using partial sequencing obtained from the hypervariable segment I (HVS-I) and coding regions 8,001-9,000 and 9.801–10,900 and 85 complete mtDNA genome sequences. Bayesian analysis of population structure was used to examine their relationship with over 3662 individuals representing indigenous groups of Taiwan, continental East Asia, Japan, and Island Southeast Asia. The whole analysis identified 278 haplotypes. Complete genomes revealed 62 novel subhaplogroups, of which 31 were exclusive to Taiwan. Estimates of coalescence times of all subhaplogroups showed peaks of diversification greater than 5.0 kya, likely characterizing gene flow from continental East Asian groups but not excluding in situ Taiwanese ancestry. Furthermore, a significant number of clades exclusive to non-Austronesian speakers of Taiwan (NAN_Tw) showed coalescence peaks between 1.0 and 2.6 kya, suggesting possible late Neolithic to early metal age settlements of NAN_Tw and local expansion in Taiwan.

## Population genetics: Mitochondrial DNA reveals ancestry of non-Austronesian-speaking Taiwanese

The first comprehensive analysis of mitochondrial DNA in non-Austronesian-speaking people from Taiwan points to an early settlement of the island by Mainland Southeast Asians in the late Neolithic period, followed by periods of isolation and genetic expansion. Marie Lin and Jean Trejaut from Mackay Memorial Hospital, Taipei, Taiwan, characterized mitochondrial DNA segments from 672 speakers of Sinitic or so-called ‘Chinese languages’ living in Taiwanese cities and lowland rural regions. The researchers also fully sequenced the entire mitochondrial genomes of 85 of these individuals. They identified numerous different genetic lineages, including many unique to Taiwan, that dated back millennia, perhaps as far as around 5000 years ago. The results point to prehistoric settlements and periods of local expansion among the ancestors of today’s non-Austronesian speakers long before groups arrived from China in modern times.

## Introduction

Taiwan is ethnically diverse^1^ and approximately 23.5 million people live there. While Mandarin is the official language, over 16 languages are spoken, principally belonging to two linguistic families: Austronesian (AN) and Sino-Tibetan (Mandarin, Minnan, and Hakka). During the last glacial maximum (LGM) over 20,000 years ago (20 kya), ice sheets covered much of the Northern Hemisphere, the sea level was low, and Taiwan still connected the East Asian continent. The discovery of Paleolithic artifacts of a tool industry at the “Changbin culture” site in Taitung and ancient human remains (bones and teeth of the Tso-Chen Man) in present-day southeast Tainan allowed archaeologists to show that Taiwan entered the Paleolithic era approximately 28 kya^2–4^.

**Fig. 1:**
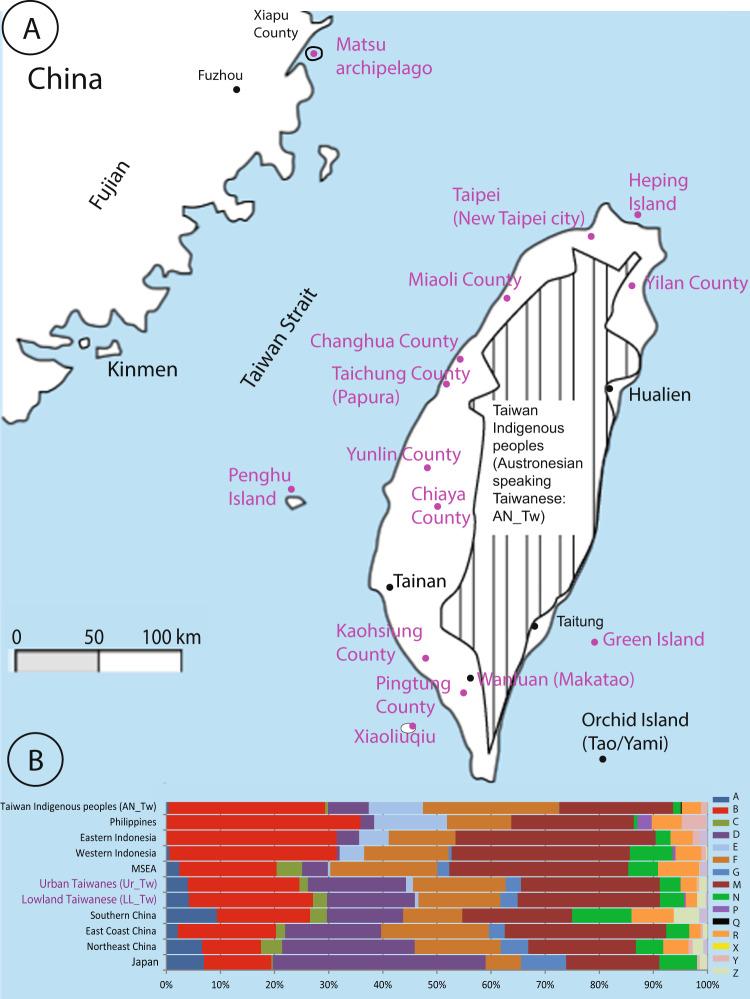
Location of Non-Austronesian-speaking groups of Taiwan (NAN_TW). **A** NAN_TW are dots colored pink; they comprise lowland and offshore Taiwanese groups (LL_Tw) and urban Taiwanese (Ur_Tw, composed of Minnan and Hakka individuals from New Taipei city, Kaohsiung city and Neipu Township in Pingtung County). The crosshatched area denotes locations of the Austronesian-speaking indigenous people of Taiwan (AN_Tw). **B** Basal haplogroup sharing across eleven groups. Detailed haplogroup frequencies are shown in Supplementary Table S1.

The rising temperature toward the late Paleolithic era resulted in the rapid receding of ice and rising sea level to approximately 100 meters. Most locations in western Taiwan, a lowland region with diverse plains and hilly landscapes (Fig. 1), were once coastal locations until the sea level lowered to the present-day coastline. Along with climate change, the birth of agriculture, the abundance of food, organized communities, human migrations, and the progress of navigation began^5^.

The Neolithic era of Taiwan (4,000 BC) saw the first settlement of sedentary groups^3^ from eastern South China. These groups spoke mutually intelligible dialects derived from Proto-Austronesian (PAN)^6^ and settled separately all over the island. Isolation by distance or geographic landscape caused cultures and languages to drift apart. Today, linguists recognize nine distinct local branches of PAN. These are still actively used by the indigenous peoples of Taiwan (or Austronesian speakers of Taiwan, AN_Tw), who represent only 2.3% of the Island population^1,7^. A tenth branch of the Austronesian language family, the Malayo Polynesian^1,7^, developed in the Batanes Islands (Ivatan) and Luzon in the Philippines (2200 BC)^7,8^. It rapidly divided further into subbranches found nearby in Malaysia, North Vietnam, Indonesia, the Bismarck Archipelago, and the Pacific Ocean, then reached New Zealand less than a thousand years ago, and finally Madagascar in the Indian Ocean^8^.

This linguistic diaspora was most likely also the result of trading networks. It started as early as 2800–2200 BC with the nearby Penghu Islands (Pescadores Islands) in the Taiwan Strait because of the need of the late Tabenkeng (or TPK) people in Taiwan to obtain material from the Penghu Island quarries to build their basalt adzes^8^.

Along with these trading networks, new arrivals soon introduced millet and wet rice agriculture^8–11^. The improved food production contributed to population density. Ultimately, from 500 BC to 500 AD, these trading networks included the Philippines, mainland Southeast Asia, India, and even widespread trades within Taiwan. Imports, such as metal technology, all came from mainland Southeast Asia (MSEA). Fujian, Guangdong and Taiwan were not yet part of the Han, and no contact with “Han-China” existed yet^12^. A millennium later, Taiwan became part of an even more active East and Southeast Asia maritime network involving Fujian merchants, pirates, and Japanese traders and spread further in Southeast Asia (SEA) and MSEA^13^. Along with these traders, fishers from the China Sea and the Taiwan Strait often visited Matsu, Penghu Island, and the west coast of Taiwan, where some settled^13^.

The end of six millennia of a uniquely Austronesian-speaking place and the beginning of Taiwan’s recorded history started with the Dutch in 1,624. To expand their deerskin trade, they established a colonial regime among more than 130 lowland villages of Indigenous peoples. The Spanish later established a base in northern Taiwan^14^. From 1895 to 1945, the Japanese instituted fifty years of colonial rule. Interestingly, the elements of Japanese culture are still part of Taiwan’s everyday life.

In the past few decades, molecular geneticists have characterized the genetic diversity of most Asian populations by first analyzing the highly polymorphic autosomal HLA system^15^. These studies allowed scientists to use the polymorphism of East Asian populations to investigate a possible relationship between genetics and Austronesian language families in Southeast Asia^16^. Then, geneticists used the maternal mitochondrial DNA (mtDNA) and paternal Y chromosomal (Y-SNP and Y-STR) gene systems. These uni-parental gene systems never recombine; they are exclusively inherited maternally or paternally. In addition to the linguistic relationship with genetics, many studies have used these gene systems to characterize gene flow, mixture, and the origin of many autochthonous groups^17,18^ and have more recently used DNA from ancient human remains to retrace the origin of archaic humans in East Asia^19,20^.

To provide more insights into the distribution and evolutionary history of East Asian populations, several research groups compared high-coverage whole-genome sequencing and genome-wide SNP autosomal data of East Asians, Taiwanese Han, and indigenous people of Taiwan and Island Southeast Asia^21–23^. The three research groups^21–23^ showed that Taiwanese Han had a low level of admixture with autochthonous Taiwanese and were descendants of several lines of ancestry spatially distributed throughout continental Asia. Their finding was generally consistent with the self-information given by the participants. Importantly, they produced documentation about known disease risk variants potentially affecting specific groups^21^.

However, only a few complete mtDNA sequences from non-Austronesian-speaking Taiwanese (NAN_Tw) people are available in the literature^19^. Rapid advances in complete genome sequencing also allow sequencing and analysis of large numbers of individuals. We used these methods to obtain 672 partial mitochondrial genomes of unrelated NAN_Tw individuals from main urban centers (Ur_Tw) and lowland locations all over the island (LL_Tw). Of these, 84 were used for complete mitochondrial genome sequencing. This study proposes to analyze the human demography, genetic diversity, and matrilineal ancestry of NAN_Tw (Ur_Tw and LL_Tw) individuals to characterize possible late Neolithic settlements and local expansion of non-Austronesian speakers in Taiwan. In doing so, this project is the first comprehensive mitochondrial DNA analysis of non-Austronesian-speaking Taiwanese individuals.

## Materials and methods

Whole blood or buccal swabs were collected by L.M. from 622 (499 LL_Tw and 123 Ur_Tw) healthy, unrelated NAN_Tw individuals (Table 1, Fig. 1, Supplementary Table S1) whose grandparents were from the same locality. We performed DNA preparation using the QIAamp DNA Blood Mini Kit (Qiagen Inc. Chatsworth, California, United States) according to the procedure recommended by the manufacturer with minor adjustments. Complete mtDNA genomic and partial sequencing (HVS-I at nps 16001 to 16569 and coding regions at nps 8001 to 9000 and 9801 to 10900) were performed on both strands using the Perkin-Elmer/Applied Biosystems Division (ABI Taiwan) DyeDeoxy Terminator Cycle Sequencing Kit (Foster City, California, United States) according to the recommendations of the manufacturer on an automated DNA sequencer (ABI Model 377). With samples from the literature (Table 1 and Supplementary Table 1), the NAN_Tw group comprised 767 individuals (i.e., 218 Ur_Tw and 499 LL_Tw, including offshore locations from this study and 50 LL_Tw from Ko et al. 2014)^19^. Individuals from the Matsu archipelago (*n* = 50)^24^ and the Fujian province (*n* = 148)^25,26^ represented East Coast China. Complete mitochondrial genomes from the literature included Vietnam (*n* = 609), Thailand (*n* = 560), Japan (*n* = 664), Northeast China (*n* = 257), Southeast China (*n* = 65), Malaysia (*n* = 87), and 260 individuals from the Philippines^19,27–32^ (Table 1).

**Table 1:** Sample data.

Population Samples	Regions/tribes	Township	Code	Groups	Sample size	Linguistic affiliation	(Taiwan) Referred as Makatao in the text	References
Changhua	Changhua County (Taiwan)	FangYuan township	CH	LL_Tw	71	Sinitic	Partial and complete Sequences	This study
ChiaYi	ChiaYi County (Taiwan)	Budai township	CYI	LL_Tw	23	Sinitic	Partial and complete Sequences	This study
Green Island	Taidong County (Taiwan)	Green Island	GI	LL_Tw	25	Sinitic	Partial and complete Sequences	This study
Heping	Keelung County (Taiwan)	Heping Island	HP	LL_Tw	10	Sinitic	Partial and complete Sequences	This study
Kaohsiung	Kaohsiung County (Taiwan)	Hunei township	KH	LL_Tw	71	Sinitic	Partial and complete Sequences	This study
Miaoli	Miaoli County (Taiwan)	Tongxiao township	Miao	LL_Tw	18	Sinitic	Partial and complete Sequences	This study
Papura	Taichung County (Taiwan)	Qingshui District	Pa	LL_Tw	38	Sinitic	Partial and complete Sequences	This study
Penghu	Penghu County (Taiwan)	Magong City	Penghu	LL_Tw	47	Sinitic	Partial and complete Sequences	This study
Pingtung	Pingtung county (Taiwan)	Wandan township	PT	LL_Tw	12	Sinitic	Partial and complete Sequences	This study
Taipei (Minnan and Hakka)	New Taipei City (Taiwan)	Wugo and Tamsui	NTP	Ur_Tw	123	Sinitic	Partial and complete Sequences	This study
Xiaoliuqiu	Kaohsiung County (Taiwan)	Liuqiu island	LC	LL_Tw	11	Sinitic	Partial and complete Sequences	This study
Yilan	Yilan County (Taiwan)	Yilan City	IL	LL_Tw	24	Sinitic	Partial and complete Sequences	This study
Yunlin	Yunlin County (Taiwan)	Shuilin	YL	LL_Tw	149	Sinitic	Partial and complete Sequences	This study
Matsu	LienChiang County (Taiwan)	Nangan island	MS	East Coast of China	50	Sinitic	Partial and complete Sequences	^24^
Makatao	Pingtung county (Taiwan)	Wanluan Township	Mak	LL_Tw	50	Sinitic	complete Sequences	^19^
Hakka_Ko^a^	Pingtung County (Taiwan)	Neipu Township	Hak	UR_Tw	45	Sinitic	complete Sequences	^19^
Minnan_Ko^a^	Kaohsiung County (Taiwan)	Kaohsiung city	Min	UR_Tw	50	Sinitic	complete Sequences	^19^
Fujian Province (China)	Fujian province (China)	Miscellaneous samples	Fj	East Coast of China	148	Sinitic	Partial and complete Sequences	^25,26^
Japan	Tokyo, Nagoya	Tokyo, Nagoya	Japan	Japan	664	Japonic	complete Sequences	^32^
Northeast China	Beijing, Henan, and Liaoning	Beijing, Henan, and Liaoning	NE_China	NE_China	257	Sinitic	complete Sequences	^57,58,59^
South China	Guangdong	See original Reference	Sth_China	Sth_China	65	Sinitic	complete Sequences	^57,60^
Vietnam	Hanoi; Northeast and Central highlands	See original Reference	Vietnam	MSEA	603	Austroasiatic	complete Sequences	^28^
Thailand	Mon; Yuan; Lue; Khuen; Karen	See original Reference	Thailand	MSEA	560	Kra-Dai	complete Sequences	^30^
Malaysia	Bidayu, Jehai, Selatar, Temuan	See original Reference	Malaysia	MSEA	86	Malayo Polynesian	complete Sequences	^29^
Indonesia (East)	Sulaweisi, Timor, Maluku	See original Reference	East_Ind	ISEA	72	Malayo Polynesian	complete Sequences	^61,49,62^
Indonesia (West)	Borneo, Java, Sumatra	See original Reference	West_Ind	ISEA	326	Malayo Polynesian	complete Sequences	^61,49,62^
Philippines	Luzon, Visayas, Mindanao	See original Reference	Ph	ISEA	260	Malayo Polynesian	complete Sequences	^27^
Austronesian-speaking Taiwanese	All tribes as in Ko 2014	See original Reference	AN_Tw	AN_Tw	426	Austronesian	complete Sequences	^19,60,63–65^

Heping Island, Green Island and Xiauliuqiu are included in the lowland Taiwanese group. Matsu is considered East Coast China.

*MSEA* Mainland Southeast Asia, *AN_Tw* Austronesian-Speaking Taiwanese (Taiwan Indigenous Peoples), *ISEA* Island Southeast Asia, *Sth_China* South China, *NE_China* Northeast China, *SE_Coast of China* Southeast Coast of China, *LL_Tw* Lowland Taiwanese, *AN_Tw* Austronesian-Speaking Taiwanese (Taiwan Indigenous People).

^a^We attached the author name to the Minnan and Hakka groups to differentiate them from the Taipei group.

Sequence regions (coding regions nps 8001 to 9000 and 9801 to 10900, and HVS-I region nps 16001 to 16519) from this study and samples from the literature were concatenated and aligned. Assignation of mtDNA to haplogroups of the samples was conducted using HaploGrep 2.0 software along with PhyloTree build 17^33^. Ambiguous haplogroup assignments were resolved using complete mtDNA genome sequencing (Supplementary Table S1).

### Statistical analyses

#### Population structure and demographic history

The program Arlequin 3.5^34^ was used to determine intrapopulation haplotype frequencies, pairwise fixation indices (*Fst*), gene diversity (H), nucleotide diversity (π), and the gene flow exchanged between groups (M = N_e_m) (Table 3). The analysis was performed using the aligned concatenated coding regions at nps 8001 to 9000 and 9801 to 10900 and HVS-I at nps 16051 to 16400 from this study and the matching regions from complete genome datasets obtained from the literature (Table 1).

Gene contributions from two putative parental populations (Han, and Austronesian speaker of Taiwan) as well as the unshared gene portion in the hybrid populations were inferred using the analysis of shared haplogroups between populations^35^ (Supplementary Table S2). The putative Han parental group comprised Northeast, East and South China, and the putative Austronesian group comprised all AN_Tw haplogroups not shared with the putative Han parental group (Supplementary Table S1).

**Fig. 2:**
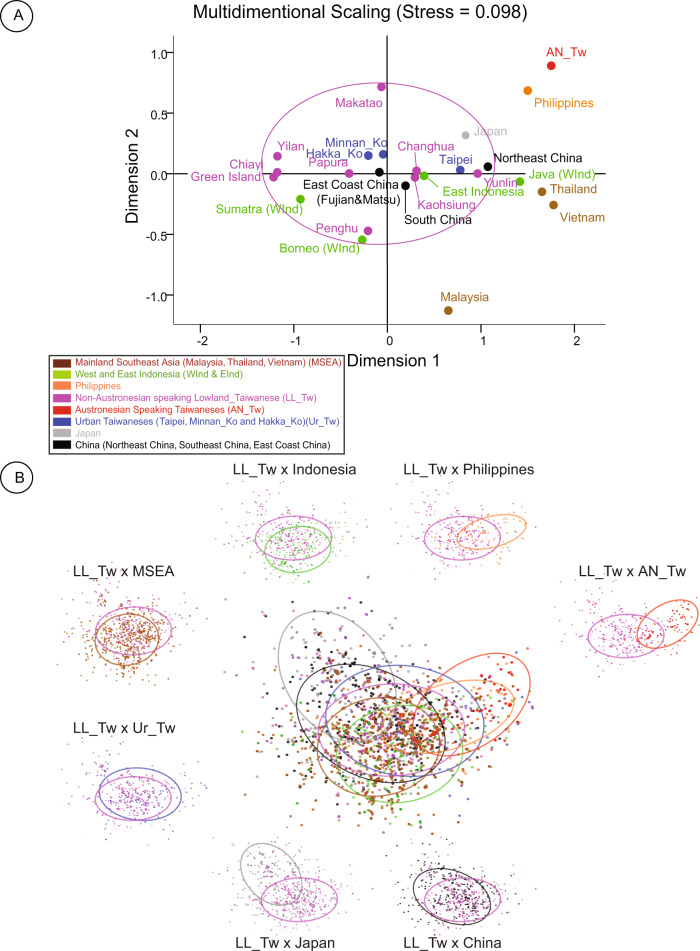
Multidimensional Scaling (MDS) and Discriminant Analysis of Principal component (DAPC). **A** MDS of population groups: Distances are Fst distances calculated from nucleotide differences. Groups with fewer than 20 individuals were removed. **B** DAPC plots: Each dot represents an individual. Groups are inertia ellipses color-coded as in the MDS plot. Each group around the central DAPC plot is compared to the LL_Tw. MSEA, Ur_Tw and China showed the most affinity with LL_Tw. Note: East Coast China includes individuals from the Fujian province of China and the Matsu Archipelago.

Past population expansions were tested using Tajima’s D and Fu’s Fs neutrality tests^36,37^ with *p* values generated using 1,000 coalescent simulations under a model of selective neutrality. Multidimensional scaling analysis (MDS) of the groups was performed with SPSS software version 17^38^ using pairwise fixation indices *Fst* (Arlequin 3.5)^34^ (Fig. 2A). To explore the genetic variability within individuals, we applied a discriminant analysis of principal components (DAPC) using R software with the adegenet package v. 3.3.1^39^.

#### Coalescence time estimates

mtPhyl software (https://sites.google.com/site/mtphyl/) was used to construct a phylogenetic tree comprising 77 unique complete mtDNA sequences from NAN_Tw. Coalescence time estimates of all clades comprising more than three subbranches, including literature data (Table 1), were calculated using the ρ statistic-based method^40^ with Gompert’s function for the complete mtDNA genome, as follows:

$$\left[ {{{{\mathrm{Time}}}}\left( {{{{\mathrm{ka}}}}} \right) = \left[ {{{{\mathrm{m}}}}\rho \,{{{\mathrm{exp}}}} - \left( {{{{\mathrm{exp}}}}\left[ { - 0.0263\left( {\rho + 40.28} \right)} \right]} \right)} \right]} \right.$$

where m is the substitution rate estimate of one site per 3.624 kya^41^. Furthermore, a combined substitution rate of 5.27 E^−8^ per nucleotide per year (or one substitution per 8,940 years) was used when dating the 2550 concatenated nucleotides (nps 8001 to 9000; 9801 to 10,900 and 16,051 to 16,400) used to construct median joining networks^42^. It should be noted that this rate was inferred from the combined rate of the HVS-I control region (1.602 E^−7^ substitutions per site per year, or one site per 17,343 years)^41^, and the substitution rate of the 2121 nps of coding region (3.42 E^−8^ per site per year, or one site per 13,786 years). The last was inferred from haplogroup E1 of the 8000-year-old human remains found in Liangdao^19,41^.

#### Mismatch distribution and demographic history

Determination of spatial range expansion and stationary population history were tested using mismatch distribution with Arlequin 3.5^34^ along with Harpending’s raggedness index^43^ to quantify smoothness and the sum of squared deviations (SSD) between observed and expected mismatches (Supplementary Fig. S2). Population expansion was inferred when a significant negative Fu’s Fs value and a nonsignificant SSD were obtained^43^. Furthermore, the demographic history of the population groups (Supplementary Fig. S2) was inferred using the Bayesian Skyline Plot (BSP) model with BEAST version 1.7^44^ and the following parameters: HKY model, relaxed lognormal molecular clock model, and coalescent Bayesian Skyline with 4 groups. The first 25% of the generations were discarded as burn-in. All analyses were run to achieve an effective sample size (ESS) greater than 200 for all estimated parameters; on average, runs with Markov-Chain-Monte-Carlo (MCMC) chain lengths were greater than 1 × 10^7^, and trees were sampled every 1000 generations. The effective population size for the posterior distribution of the estimated parameter values was determined using Skyline plot analysis with TRACER version 1.7.1^44^.

#### Population gene flow and ancestry sharing

The population genetic grouping was characterized using Bayesian population mixture analysis with Bayesian Analysis of Population Structure v. 6.1 (BAPS)^45^. To accurately detect the uppermost hierarchical level of structure for the BAPS analysis, we used the Evanno statistic^46^ based on the rate of change in the log marginal probability of data between successive K values. Mixture analysis with *K* = 2 to 22 was first carried out to determine the Delta K (Supplementary Fig. S7). We then used *K* = 2 to 30 to produce a heatmap showing gene flow between source and target groups using the BAPS option “Admixture of individuals based on mixture clustering” (Supplementary Fig. S3).

#### Ethics statement

All participants gave written informed consent before biosample collection for subsequent population analysis. The study was authorized by the ethical committee of the Institutional Review Board (IRB) of the Mackay Memorial Hospital in Taipei, Taiwan (# 11MMHIS180).

## Results

The study comprised 767 mtDNA samples from NAN Taiwanese individuals, often referred to as Han Taiwanese, or Minnan and Hakka. This group included thirteen lowlander/islander-Taiwanese groups (LL_Tw) and an urban Taiwanese group (Ur_Tw) composed of Minnan and Hakka residents from Kaohsiung City, Pingtung County (thereby named Minnan_Ko and Hakka_Ko), and Minnan and Hakka from Taipei^19^ (Table 1 and Fig. 1). Samples were initially screened by sequencing 2,669 nucleotide positions (nps) of the mtDNA of the coding and control (HSV-I) regions (nps 8,001 to 9,000, nps 9,801 to 10,900, and nps 16,001 to 16,569). This protocol identified 653 different haplogroups, 278 among NAN_Tw, of which 125 were shared by two Taiwan groups or more. MtDNA haplogroups were assigned using Haplogrep 2 software^47^. There were minor differences between LL_Tw and Ur_Tw. Basal haplogroups and their most representative subgroups (Fig. 1 and Supplementary Table S1) in NAN_Tw comprised haplogroups A (3.67%), B (23.34% with B4 = 16.14%, and B5 = 6.28%), C (3.28%), D (15.86% with D4 = 8.91%, D5 = 6.42%, and D6 = 0.53%), E (1.57% with E1a = 0.92%, and E2b = 0.52%), F (13.88% with F1a = 7.33%, F2 = 3.28%, F3 = 1.31%, and F4 = 0.78%), G (3.54%, with G1 = 0.92% and G2 = 2.36%), M (24.90% with M7b1, 8.54%, M7c1, 4.33%; M8, 3.66%, and M10a, 3.02%;), N9a (4.06%) and R9 (2.49%) (Fig. 1). To obtain better haplogroup assignments, the complete genome of poorly characterized haplotypes was sequenced. This procedure allowed us to define 62 novel subhaplogroups, of which 31 were exclusive to NAN_Tw (Table 2, Supplementary Fig. S1, and Supplementary Text 1). When naming a novel subhaplogroup, the exclusive nucleotide substitution closest to the tip of its phylogeny was added to the basal haplogroup name. We preferably used an SNP matching our present screening protocol and an underline for naming unreported haplogroups (e.g., M7b2a_8389) (Supplementary Fig. S1 and Table S3)^47^.

**Table 2:** Time of most recent common ancestor (TMRCA) of novel haplogroups and their origins.

	Temptative local names of Novel Haplogroups	TMRCA (kya) estimated from this study and literature complete mtDNA dataset (Table 1)	Number of complete mtDNA genomes observed in this clade (This study)	Number of partial mtDNA lineages (this study and literature data set)	Hapllogroups exclusive to NAN_Tw	Main location	Temptative origin
1	A20_16148	1 (0.1; 2.1)	2	7	Exclusive	Changhua, Yunlin	NorthEast China
2	B4a_16234	0.7(0; 1.7)	1	6	Exclusive	Kaohsiung	East Coast China (Fujian)
3	B4c1b2b_16305	3.2 (−0.1; 6.7)	1	9	Exclusive	Ur_Tw	MSEA/Taiwan
4	B4c1b2c1_16280	2.6 (−1.2; 3.8)	1	2	Exclusive	Changhua	MSEA/Taiwan
5	B4c1b2c2_10250	na < 8.84 (5;12)	1	3	Exclusive	Kaohsiung	East Coast China (Fujian), MSEA/ ISEA
6	B4h1_10581	na < 9 (5;12)	1	7	Exclusive	Changhua	East Coast China (Fujian), MSEA
7	C7a_10310	8.8 (5.3; 12.4)	1	8	Exclusive	Ur_Tw	China/MSEA
8	D4a3b_16294	7.8 (1; 15.9)	1	4	Exclusive	Ur_Tw	East Coast China (Fujian)
9	D4b1b2_16380	3.2 (−0.1; 6.7)	2	5	Exclusive	Ur_Tw, Green Island	NorthEast China/Japan
10	D4b2b2b_16274	4.3 (0.5; 8.2)	1	2	Exclusive	Yunlin	NorthEast China/Japan
11	D4b2b_10310	7.8 (1; 15.9)	1	2E	Exclusive	Yunlin	China/Japan
12	D5a2a1b1_16293	2.6 (−0.3; 5.6)	1	11	Exclusive	Penghu Island	NorthEast China/Japan
13	D5b1b2_16249	2.6 (0.6; 4.6)	1	2	Exclusive	Yunlin	China/MSEA
14	F1a1_8589	2.6 (−1.2; 6.4)	1	4	Exclusive	Papura	East Coast China (Fujian)/MSEA
15	F2_10313	2.2 (0.3; 4.1)	1	6	Exclusive	Changhua	East Coast China (Fujian)/MSEA
16	F2_8264	4.6 (0; 9.3)	1	10	Exclusive	Yilan	East Coast China (Fujian)/MSEA
17	F4a2_16243	6.7 (2.4; 11.2)	1	2	Exclusive	Penghu	NorthEast China
18	F4b1_8665	4.5 (1.8; 7.2)	1	1	Exclusive	Changhua	Fujian, AN_Tw, ISEA
19	M7b1a1_11659	na < 10.3 (7.3; 13.4)	1	1	Exclusive	Green Island	East Coast China (Fujian)
20	M7b1a1a1b_16162	na <3.6 (0.6; 6.8)	1	4	Exclusive	Kaohsiung	China/MSEA
21	M7b1a1a_8406	1.9 (−0.9; 4.8)	1	3	Exclusive	Kaohsiung	China/MSEA
22	M7b2a_8389	2.6 (−0.5; 5.7)	1	5	Exclusive	Yilan	East Coast China/MSEA/ISEA
23	M7c1a3a_469	2.6 (-1; 6.2)	3	3	vxclusive	Changhua, Yilan, Yunlin	NorthEast China/Japan
24	M7c1a3a_1664	na < 5 (2.4; 7.8)	1	1	Exclusive	Matsu	NorthEast China/Japan
25	M7c1a3_3027	1.9 (−0.3; 4.2)	2	2	Exclusive	Ur_Tw, Chiayi	NorthEast China/Japan
26	M7c1a4a_16173	3.7 (1.2; 6.3)	2	11	Exclusive	Penghu Island, Miaoli	China/ISEA
27	M7c1a4b(16295 < -)	5.2 (−0.2; 10.8)	1	6	Exclusive	Ur_Tw	AN_Tw
28	M8a2_8503	1.3 (−1.2; 3.8)	1	3	Exclusive	Yunlin	China
29	M8a2a1_8410	1.7 (−1.6; 5.1)	1	3	Exclusive	Yilan	China
30	N10_10586	1.3 (−1.2; 3.8)	1	2	Exclusive	Chiayi	ISEA
31	Z4_16248	1.3 (−1.2; 3.8)	1	5	Exclusive	Yunlin	NorthEast China (Fujian)
32	B4a5_8730	1.0 (0; 3.3)	1	2		Yunlin	East Coast China (Fujian)
33	B4b1a2a_16309	4.8 (0.3; 9.3)	1	28		Changhua	MSEA, East Coast China (Fujian)
34	B4c1b2a_16242	3.8 (−0.1; 6.7)	2	15		Changhua	East Coast China (Fujian), MSEA/ ISEA
35	B5a_16260/274	4.3 (−0.2; 9)	1	8		Ur_Tw	East Coast China (Fujian), MSEA, ISEA
36	B6a1a_16051	15.0 (7;23)	1	6		Kinmen	East Coast China (Fujian)/ ISEA/MSEA
37	D4a3b2_16278	1.6 (0.3; 3)	2	55		Ur_Tw, Yunlin	East Coast China (Fujian)
38	F1a1_10463	8.9 (3.3; 14.7)	1	21		Changhua	East Coast China (Fujian)/MSEA
39	F1a1_8265	na < 12.2 (5; 19.7)	1	1		Matsu	East Coast China (Fujian)
40	F1a1a_16311	7 (0.7; 13.6)	2	18		Ur_Tw, Matsu	East Coast China (Fujian)/MSEA
41	F1a3b	10.8 (3.6; 18.3)	1	38		Kaohsiung	East Coast China (Fujian)
42	F1a_10604	8.3 (4.5; 12.2)	1	19		Ur_Tw	NorthEast China
43	F2a1_16318	2.6 (0.6; 4.6)	1	18		Matsu	East Coast China (Fujian)
44	F2b1	1.3 (−1.2; 3.8)	1	45		Kinmen	East Coast China (Fujian)/MSEA
45	M7b1a1f	10.3 (7.3; 13.4)	1	65		Yunlin	East Coast China (Fujian)/MSEA
46	M7b1a1h_16400	na < 7.9 (2.7; 13.3)	1	13		Chiayi	East Coast China (Fujian)/MSEA
47	M7c1_16104	na	1	2		Yunlin	East Coast China/MSEA/ISEA
48	M7c1a1a_9833	2.6 (−1; 6.2)	1	4		Matsu	South China/Japan
49	M7b1a1_12361	na < 10.3 (7.3; 13.4)	1	1		Green Island	China/Japan/MSEA
50	M7c1b2b_8404	3.2 (0.4; 6.1)	1	14		Matsu	East Coast China (Fujian)
51	M8a2a1_16390	7.2 (3.4; 11.1)	1	18		Ur_Tw	MSEA
52	M8a2a1_8245	1.3 (−1.2; 3.8)	2	14		Yilan, Penghu Island	East Coast China (Fujian)
53	M10_16256^a^	1.7 (−0.7; 4.1)	1	15		Yilan	MSEA/Vietnam
54	M10a1a_10245	6.5 (2.3; 10.9)	4	49		Ur_Tw, Yilan, Yunlin, Penghu Island	China
55	M74b_10648^a^	8.6 (2.3; 15)	1	2		Ur_Tw	West Indonesia/MSEA
56	N9a3_10321	2.6 (0.5; 4.7)	3	15		Matsu, Penghu Island, Yunlin	East Coast China (Fujian), South China
57	N9a4b1_16091	5.7 (1.9; 9.5)	1	5		Yilan	North and South China
58	N9a9_16390	6.5 (0.8; 12.5)	2	11		Green Island	NorthEast China, Japan
59	R9b1a2b_16239	na < 11.5 (5.7; 17.6)	1	3		Ur_Tw	East Coast China (Fujian)/MSEA
60	R9b1a3	29,6 ± 9.9	1	16		Yunlin	NorthEast China/MSEA
61	R9c_10403	6.7 (1.3; 12.2)	2	8		Yunlin	East Coast China (Fujian)/MSEA/ISEA
62	Y1b1	3.6 (0.6; 6.8)	1	3		Matsu	China

Haplougroups followed by an “underlign” and a variant mutation are novel haplogroups/lineages (i.e. not reported in Phylotree 17).

*TMRCA* Time of most recent common ancestor, *kya* Thousand years, *na* Not applicable (not enough lineagse to estimate age).

^a^reported in other studies but not in Phylotree 17.

### Genetic structure

Gene diversity indices calculated from haplogroup frequencies generally showed higher values in mainland China, LL_Tw, Thailand, and Vietnam (H > 0.841) groups than in Austronesian-speaking groups, the Philippines, Indonesia, Malaysia, and AN_Tw (Table 3, Supplementary Table S1). To the exception of unreliable results obtained from groups with a small sample size (i.e., Pingtung *n* = 12, Heping Island = 10, and Xiaoliuqiu *n* = 11), the nucleotide diversity indices (π) were evenly distributed throughout Taiwan and showed no distinct differences among groups in continental Asia (π = 0.004 to 0.006) (Table 3). Similarly, except for Pingtung showing a mean number of pairwise differences (MNPd = 9.48), MNPd in LL_Tw and Ur_Tw were within the range seen in continental Asia (MNPd = 14.34 to 19.96) (Table 3 and Supplementary Fig. S2).

**Table 3:** Molecular Diversity.

	Population samples	Sample_size (n)	Number of haplotypes (k)	k/n	Number of polymorphic sites	Haplogroup diversity (Ĥ ± SE)	Nucleotide diversity (π ± SE)	Tajima’s D	Fu’s FS test	Sum of Square differences (SSD)	Mean number of pairwise differences (MNPd)	Geneflow exchanged between groups (M = N_e_m)	Raggedness index
Taiwan Dataset (NAN_Tw)	Changhua (LL_Tw)	71	66	0.93	157	0.8743 ± 0.0012	0.006 ± 0.003	−1.90**	−24.14***	0.003	17.07	10581.82	0.002
	Chiayi (LL_Tw)	23	23	1.00	78	0.8744 ± 0.0018	0.005 ± 0.002	−1.37	−12.25***	0.018	14.34	5457.94	0.013
	Green_Island (LL_Tw)	25	18	0.72	68	0.8449 ± 0.0031	0.006 ± 0.003	−0.75	−12.72**	0.011	16.66	32.59	0.022
	Heping_Island (LL_Tw)	10	8	0.80	40	0.6954 ± 0.0245	0.005 ± 0.003	−0.23	−2.48	0.047	14.62	15.50	0.079
	Kaohsiung (LL_Tw)	71	63	0.89	154	0.8677 ± 0.0019	0.006 ± 0.003	−1.88**	−24.13***	0.005	17.41	712.30	0.003
	Miaoli (LL_Tw)	18	17	0.94	73	0.7705 ± 0.0095	0.006 ± 0.003	−1.34	−7.11**	0.014	16.40	445.13	0.003
	Papura (LL_Tw)	38	36	0.95	102	0.8322 ± 0.0048	0.005 ± 0.002	−1.54*	−24.29***	0.002	15.27	615.56	0.003
	Penghu Islan (LL_Tw)	47	40	0.85	110	0.8414 ± 0.0031	0.005 ± 0.002	−1.63*	−24.27***	0.004	15.71	145.58	0.004
	PingTong (LL_Tw)	12	12	1.00	42	0.7761 ± 0.0007	0.003 ± 0.001	−1.45	−5.14**	0.020	9.48	5136.77	0.058
	Taipei (Ur_Tw)	98	83	0.85	154	0.8733 ± 0.0017	0.005 ± 0.002	−1.83**	−24.10***	0.003	15.33	631.66	0.001
	Xiaoliuqiu (LL_Tw)	11	11	1.00	74	0.7521 ± 0.0000	0.007 ± 0.004	−1.32	−2.27	0.018	19.96	8687.13	0.033
	YIlan (LL_Tw)	25	20	0.80	79	0.7975 ± 0.0062	0.005 ± 0.003	−1.25	−13.12**	0.009	15.98	45.53	0.012
	Yunlin (LL_Tw)	149	115	0.77	185	0.8866 ± 0.0014	0.005 ± 0.002	−1.92***	−23.92**	0.003	15.98	340.13	0.002
Literature Dataset	Matsu (East Coast China)	50	47	0.94	125	0.8449 ± 0.0031	0.005 ± 0.002	−1.72*	−24.28***	0.041***	15.03	963.27	0.016
	Fujian (East Coast China)	148	121	0.82	184	0.8744 ± 0.0018	0.005 ± 0.002	−2.00**	−23.97***	0.002	14.90	794.96	0.002
	East Coast China (Fujian & Matsu)	198	159	0.80	213	0.9181 ± 0.0004	0.005 ± 0.002	−2.04**−	23.85**	0.003	14.90	795.47	0.001
	East Indonesia (ISEA)	72	55	0.76	127	0.8230 ± 0.0048	0.006 ± 0.003	−1.66*	−24.15***	0.002	14.90	138.04	0.003
	Hakka_Ko (Ur_Tw)	45	40	0.89	114	0.8290 ± 0.0049	0.006 ± 0.003	−1.80**	−24.26***	0.007	16.11	340.39	0.003
	Japan (NE_Asia)	664	375	0.56	315	0.9037 ± 0.0008	0.004 ± 0.002	−2.15***	−23.60**	0.002	12.12	124.91	0.001
	Makatao (LL_Tw)	50	36	0.72	106	0.8123 ± 0.0056	0.005 ± 0.002	−1.60*	−24.28***	0.005	15.02	64.35	0.005
	Malaysia (MSEA)	86	32	0.37	92	0.6862 ± 0.0186	0.004 ± 0.002	−1.096	−24.31***	0.007	12.24	11.09	0.011
	Minnan_Ko (Ur_Tw)	50	48	0.96	112	0.8500 ± 0.0027	0.005 ± 0.002	−1.79*	−24.29***	0.070***	14.77	6328.73	0.004
	Northeast China (NE_Asia)	257	250	0.97	280	0.9037 ± 0.0008	0.005 ± 0.002	−2.17***	−23.78**	0.021***	14.26	1001.74	0.001
	Philippines (ISEA)	260	114	0.44	222	0.7706 ± 0.0078	0.006 ± 0.003	−1.79**	−23.66**	0.005	17.70	12.83	0.002
	South China (Sth_China)	65	60	0.92	133	0.8743 ± 0.0012	0.005 ± 0.002	−1.75*	−24.29***	0.001	13.84	269.88	0.003
	Taiwan Indigenous peoples (AN_Tw)	426	128	0.30	164	0.8154 ± 0.0042	0.006 ± 0.003	−1.42**	−23.53**	0.004	16.24	34.70	0.003
	Thailand (MSEA)	560	333	0.59	326	0.8667 ± 0.0020	0.004 ± 0.002	−2.18***	−23.61**	0.001	12.68	156.91	0.002
	Vietnam (MSEA)	603	260	0.43	280	0.8625 ± 0.0020	0.006 ± 0.003	−1.95**	−23.43*	0.006	16.82	145.19	0.001
	West Indonesia (ISEA)	326	227	0.70	259	0.8132 ± 0.0047	0.006 ± 0.003	−2.05***	−23.60*	0.004	16.84	197.46	0.001

Complete mtDNA genomes from the litterature (Table 1) have been adjusted to match the partial sequence of the Taiwan dataset. Statistics were undertaken with Arlequin software using HVS-I (nps 16006 to 16569), and coding regions (nps 8001 to 9000 and 9801 to 10900).

*Ur_Tw* Urban Taiwanese (Taipei, Minnan and Hakka), *LL_Tw* (Lowland Taiwanese), *MSEA* Mainland Southeast Asia, *ISEA* Island Southeast Asia, *NE_Asia* Northeast Asia, *Sth_China* South China, *AN_Tw* Austronesian-speaking Taiwanese, *H* haplogroup/gene diversity (Nei M. Molecular evolutionary genetics. New York: Columbia University Press; 1987), π nucleotide diversity, *MNPd* mean pairwise difference, *n* sample size, *M* geneflow exchanged between demes, SE standard error.

*p ≤ 0.02, **p ≤ 0.01, ***p ≤ 0.001.

The gene flow (*M* = *N*_e_*m*) exchanged between groups (Table 3) is affected by the effective population size, *N*_e,_ and the migration rate *m*. It is a measure of spatial expansion indicating how a population becomes subdivided when mating preferentially with neighbor groups^48^. Indigenous groups such as AN_Tw, Malaysians & Philippines tend to have a smaller *N*_e_; thus, a smaller *M* is expected when compared to NAN_Tw. As expected, the low gene flow (*M*), shown with AN_Tw indigenous groups (*M* = 14.09), contrasts with the high to very high gene flow (*M* = 145.55 to 10781) shown with NAN_Tw (Table 3). However, the comparatively low intragroup gene flow in Green Island, Heping Island, and Yilan (*M* = 27.94 to 45.53) suggests that these groups had low *N*_e_ and likely remained relatively homogeneous, isolated and had a low migration rate (low *m*). We see a similar pattern for the Philippines and Malaysia (*M* = 12.8 and 11, respectively). A significant negative Tajima’s D value (*p* < 0.01) was seen in all NAN_Tw (LL_Tw and Ur_Tw) groups with a sample size greater than 20 (Table 3). These results suggest sudden expansion. However, to be adopted, these results need support by the combination of a significant and more powerful Fu’s Fs negative test (*p* < 0.01) along with a nonsignificant sum of square difference test (SSD, *p* > 0.02)^43^, as shown in mismatch distribution analysis (Supplementary Fig. S2, Columns 1 and 3). Although most mismatch plots appeared multimodal, none of the raggedness indices (Ri) was significantly different from the expectation. The NAN_Tw Bayesian Skyline distribution plots (Supplementary Fig. S2, Columns 2 and 4) show the effective population size (*y*-axis) as a function of time (*x*-axis). All groups show a higher effective population size (*N*_e_) presently than in the past. Although many groups do not show expansion in the last few 1000 years, they show expansion until the last two to three thousand years BP. For example, while Fujian, Taipei, Changhua and Kaohsiung show an increase in *N*_e_ 30 times greater at present than in the past, smaller groups such as Green Island and the Matsu archipelago show population expansion to a much lower level (8 times). Similarly, except for small island groups such as Matsu, Heping, and Green Island showing a very low effective population size (*N*_e_ = 250), all other groups showed an *N*_e_ ranging between 1700 and 2500. Interestingly, Xiaoliuqiu shows a stationary effective population size. This is not surprising, as this island is very small (2 km wide). Its population often changed and never reached more than a few hundred individuals. However, caution should be taken when analyzing groups with a sample size of less than 20 individuals (Xiaoliuqiu, Heping, and Pingtung).

### Mixture between groups

Inference of haplogroup contributions from putative parental populations was obtained using the analysis of haplogroup proportion from molecular data^35^. We pooled the Fujian, Northeast Asia, and South China groups to constitute a putative Han parental gene source. For the ancestral Austronesian putative gene source, we pooled all Taiwan indigenous peoples (AN_Tw) and kept all exclusive haplogroups (i.e., haplogroups not shared with the Han putative haplogroups) (Supplementary Table S1). When estimating the mixture (Supplementary Table S2), the percentage of unshared haplogroups (Supplementary Table S2, third column) may represent gene flow from other regions (i.e., MSEA or ISEA). Alternatively, it can represent within-group genetic variation resulting from a long period of isolation. Interestingly, on average, LL_Tw and Ur_Tw showed similar profiles, with 23.86% and 20.07% of haplogroups shared with Austronesian indigenous peoples, 33.27% to 34.27% shared with Han peoples, and 42.87% and 45.65% shared exclusive haplogroups (or haplogroups from an undefined source).

### Diversity and demographic history

#### Multidimensional Scaling (MDS, Fig. 2A)

In the following, the East Coast of China is represented by Fujian (individuals from Xiapu County in China), self-declared individuals from the Fujian province who moved to Taiwan, and Matsu individuals^24–26^. The MDS plot (Fig. 2A) shows the affinity of the Ur_Tw groups (blue dots) with mainland China (black filled dots). The AN_Tw (red dots), the Philippines (orange dots in the upper right quadrant), and Japan (gray dots in the upper left quadrant) show much less affinity with NAN_Tw. Due to the low sample size (n < 20) of Pingtung, Miaoli, Xiaoliuqiu, and Heping, these LL_Tw groups appeared as out-groups on the MDS and were removed. The rest of the LL_Tw occupies a more central position, with Changhua, Papura, Kaohsiung, Penghu, and Yunlin showing affinity with different regions of Indonesia^49^. Last, as expected from Supplementary Table S2, Makatao with a 45% mixture with AN_Tw stands up in the same cluster as the Austronesian speakers.

#### Discriminant Analysis of Principal Components (DAPC, Fig. 2B)

DAPC (Fig. 2B) places NAN_Tw individuals (blue and purple-filled dots) in a central position. They include Ur_Tw groups (Minnan_Ko, Hakka_Ko, and Taipei in blue) and LL_Tw (purple) individuals and suggest overall homogeneity (Fig. 2B). Similarly, and consistent with previous complete human genome studies^21–23^, LL_Tw individuals show a significant ancestral relationship with mainland China (black dots). On the other hand, the Philippines (orange dots) and AN_Tw (red dots) groups show less mixture, suggesting a low amount of mixture between the NAN_Tw and AN_Tw groups and supporting results from Lo et al. 2020^23^. These results are further supported by Supplementary Table S2, which shows a 3.49% to 5.53% mixture between AN_Tw and Changhua, Miaoli, Taipei, and Minnan_Ko groups and a more than 39% mixture with Makatao and the Hakka_Ko group from Neipu Township in Pingtung County. Additionally, the closeness of AN_Tw individuals with the Philippines group supports the Out of Taiwan hypothesis (OOT)^50^. As shown in Fig. 2A, Indonesian individuals (green) and MSEA individuals (brown) show partial affinity with NAN_Tw, likely reflecting traces of an ancient trading network between Taiwan, ISEA, and MSEA. In agreement with complete human genome autosomal studies^21,23,51^, the inertia ellipse representing individuals from China (black) overlaps with Japan (gray in the upper left). Similarly, the lower end of the ellipse (Black) overlaps with mainland Southeast Asia, likely the result of the southward expansion of the Han under the previous Qin Dynasty^52^.

#### Bayesian analysis of population structure (BAPS)

##### Mixture partition using Bayesian analysis (Fig. 3A, C)

We used a visualization tool of Bayesian Analysis of Population Structure (BAPS) to analyze the genetic diversity, mixture, and relative average of the maternal ancestry between NAN_Tw and groups of East Asia^53^. We did not use tests for linguistic affiliation, as all NAN_Tw belong to the Sinitic family of languages. However, we applied the analysis to test for geographically associated clusters.

**Fig. 3:**
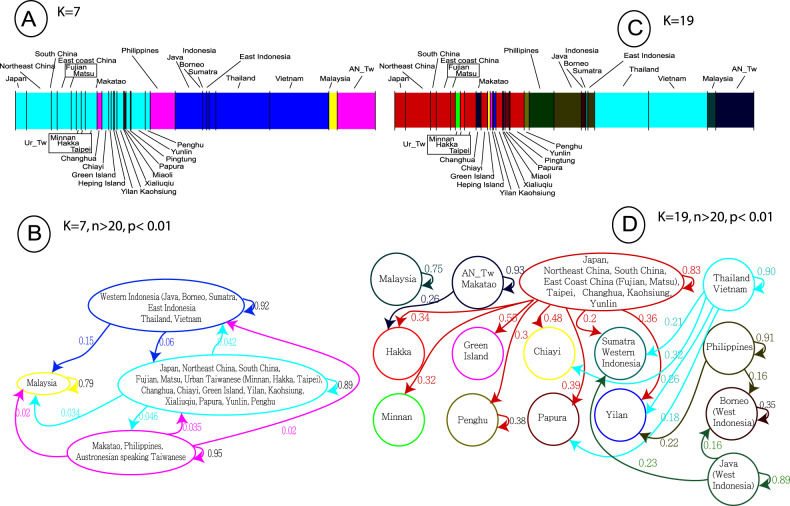
Mixture partition and map of gene flow. Plots representing the uppermost hierarchical level of structure were constructed using the cluster assignments inferred from the Delta k results (*K* = 7, and *K* = 19) shown in Supplementary Fig. S7^46^. Plots are color-coded according to their K value (i.e., *K* = 7 matches B, and *K* = 19 matches C). **A** *Mixture partition for K7 and K19*. Each vertical line represents a single individual and is colored according to the highest proportion of genetic variation assigned to each BAPS group. The order of populations is set according to the geographic orientation from north to south via Taiwan. **B**, **C** *Network of gene flow* (*p* = 0.01) for K7 and K19 and *n* > 20. Colors in (**B**) and (**C**) are associated with the mixture plot of the same *K* value in (**A**). Arrows indicate the average fraction of sequence variation obtained from the source cluster to the target cluster. Feedback arrows beside clusters indicate the fraction of gene sources arising within the BAPS group. Groups/clusters with less than 20 individuals (i.e., Heping, Miaoli, Pingtung, and Xiaoliuqiu) are not shown in the network of gene flow. Note: A complete heatmap of gene flow for all groups (*K* = 30) is shown in Supplementary Fig. S3.

Using the average log-likelihood of K (Delta K)^46^, the BAPS analysis characterized the most significant cluster variation at *K* = 7 and *K* = 19 (Supplementary Fig. S7). At *K* = 7, population clusters matched geography, except for Malaysia differentiating from MSEA and ISEA. The largest group (Light blue) includes all NAN_Tw (Fig. 3A) and China, and at *K* = 7, there is a homogeneous mixture between the groups^21,23,51^. Interestingly, this cluster lost significant homogeneity at *K* = 19 (Fig. 3A) but still includes Japan. The maternal legacy with Japan will be conserved until *K* = 23 when Japan separates from Northeast China, East Coast China, Taipei, Changhua, Kaohsiung, and Yunlin (Supplementary Fig. S6). East coast China (Fujian), Taipei, and Yunlin will show strong affinity until *K* = 28 (Supplementary Fig. S6). Finally, Makatao is the only LL_Tw group that remains separated from all other NAN_Tw. From *K* = 2 to *K* = 7, Makatao shows undifferentiated affinity with AN_Tw and the Philippines. From *K* > 9 to *K* = 23, its affinity will be restricted to AN_Tw, in agreement with Supplementary Table S2 showing Makatao with only 14.68% mixtures with putative parental Han, but 45.5% sharing with AN_Tw and 39.8% not shared with either putative group.

##### Gene flow and levels of relatedness between groups (Fig. 3B, C)

BAPS was further used to estimate the extent of gene flow and the level of relatedness between groups of more than 20 individuals. At *K* = 7 (Fig. 3B, p < 0.01), four clusters showed exclusive within-group gene sources greater than 79% (Fig. 3B). Malaysia appeared as a group that separated very early in the genetic history of mainland Southeast Asia; however, likely in the last few hundred years, it received 16% of gene flow from Thailand, Vietnam, and Indonesia. Except for Malaysia, the overall gene flow between groups at *K* = 7 did not exceed 6%. However, at *K* = 19 (Fig. 3C), clusters without exclusive within-group gene sources (i.e., NAN_Tw, and Sumatra) are targets of gene flows primarily coming from China or other groups from Taiwan. Note that at *K* = 24 (data not shown), the urban Taiwanese group shows a level of gene source within a group of 82%, likely representing the diversity from all NAN_Tw target groups or resulting from recent demographic movements of lowland individuals toward central and attractive urban centers such as Taipei or Kaohsiung.

We used *K* = 30 to construct a heatmap of pairwise gene flow between all source and target groups (Supplementary Fig. S3). Japan, Northeast China, East Coast China, and mainland Southeast Asia represent the major sources of maternal heritage for NAN_Tw, compared to the contributions from the Philippines and AN_Tw.

The gene flow from Indonesia and MSEA, inferred in Fig. 2, was not seen in Fig. 3C because of the 15% gene flow pruning applied to obtain a clear visual. However, it is seen at less than 10% in Supplementary Fig. S3, with western Indonesia (Java) appearing as a moderate source compared to MSEA. These patterns of gene sources from Indonesia and MSEA suggest possible northward movements of populations or traders from the South China seas and supplement the apparent affinity between NAN_Tw, Indonesia, the Philippines, and MSEA previously determined with the MDS and DAPC analyses (Fig. 2 and Supplementary Table S1). Furthermore, the sharing of haplogroups between western Indonesia and groups in China (10.2%) also suggests gene flows resulting from past back-and-forth migrations of traders between East China and ISEA^54^ (Supplementary Tables S1, S2).

In summary, the source populations, shown in the top section of Supplementary Fig. S3 (East Asia), represent gene pools significantly distinct from MSEA- and Austronesian-speaking groups. These populations have high within-group variation. Except for South China and East Indonesia, they receive little from elsewhere and are likely the result of long periods of local dispersal and expansion before they reached Taiwan. In contrast, except for Taipei, Yunlin, Kaohsiung, and Changhua, who belong to a major source cluster (Fig. 3B, C), all other NAN_Tw groups have low within-group variation. Most are the targets of gene flow from mainland East Asia and at a lower level from Island Southeast Asia. Finally, except for Makatao and the Hakka_Ko group from Neipu Township in Pingtung County^19^, which received gene flow from AN_Tw (45.51% and 39.82%, respectively), the general mixture distribution between AN_Tw and NAN_Tw was less than 5% (Supplementary Table S2).

## Discussion

The precise time and mode of the colonization of Taiwan by NAN groups remain a disputed issue. This study sheds light on the diversity, distribution, and origin of mtDNA haplogroups among 672 NAN individuals living in different offshore and inshore locations in Taiwan (Table 1 and Supplementary Table S1). The homogeneous distribution of high polymorphism, high genetic diversity (Table 3), and mixture throughout all groups suggests important gene flow between lowland and urban Taiwanese individuals (Fig. 2). The analysis of haplogroup sharing (Supplementary Table S2) showed little AN_Tw mixture among NAN_Tw, except for Hakka_Ko and Makatao. Interestingly, the matrilineal heritage of Makatao (45.5% sharing with AN_Tw, less than 15% with mainland Asia, and 40% of undefined sources) characterized this LL_Tw group as strongly mixed with AN_Tw (Supplementary Table S2). While strongly Sinicized, the Makatao may be a former indigenous group of Taiwan.

Recent studies^51^ used genome-wide analyses to date the common ancestor of extant Han Chinese, Korean and Japanese back to 3000 years ago and to characterize recent admixture from the surrounding populations^21,22,51^. Similar approaches^23,55^ reported that AN_Tw share ancestry with Tai-Kadai and Austroasiatic speakers and proposed that AN_Tw are genetically associated with Yangtze River Valley agriculturists. Larena et al. (2021)^20^ used 2.3 million genotypes from 118 ethnic groups in the Philippines to show that modern humans in ISEA interbred with archaic Denisovans. Genome-wide data analysis facilitates our understanding of the evolutionary history of human populations, gene flows, and their genetic diversity. However, more is still required to characterize subtle parental fingerprints within groups in more restricted geographical areas.

The screening method used in this study (using sequencing of nps 8001–9000, 9801–10,900, and the HVS-I of the control region 16,051–16,400) produced sufficient diversity to represent a statistically significant population structure of the NAN-speaking Taiwanese individuals and their relationships with other groups in East Asia. We identified 271 haplotypes in Taiwan, and complete genome sequencing characterized 62 novel mtDNA haplogroups (Table 2, Supplementary Tables S4, S5). Overall, most new variants were found close to the tips of the phylogenetic tree. Furthermore, the study revealed various maternal contributions from Northeast Asia, Fujian, South China, and MSEA individuals (Fig. 2 and Supplementary Table S1), consistent with previous complete human genome studies^21–23^. Three groups of relationships were determined (Supplementary Text S1 and Table 2): (a) A Northeast Asian group (~30%) comprising haplogroups A (A5b1b and A5b1c), C, D (D4, and D5), G, M8, M10a, and N9a1. (b) A group derived from mainland Southeast Asia and Island Southeast Asia (~60%) composed of haplogroups B (B4, and B5), E (E1a1), F (F1a, and F2), M (M7b1, and M7c1), and R9. (c) Finally, out of the 62 novel haplogroups, 16 had an immediate origin in Fujian, and 31 were seen exclusively among NAN Taiwanese (Table 2). Estimates of the time of the most recent common ancestor (TMRCA) using a single genetic system (mtDNA) may produce inconsistent estimates of the population divergence time. Accordingly, our attempts to uncover the prehistory of NAN_Tw settlement in Taiwan should be interpreted with caution. The TMRCA was calculated using complete mtDNA genomes. The TMRCA of haplogroups exclusive to Taiwan (Table 2) showed six haplogroups with a TMRCA higher than 5 kya. The remaining ranged from 1.0 to 4.0 kya. Interestingly, 14 haplogroups exclusive to NAN_Tw showed an age range of 1.0 to 2.6 kya, suggesting long isolation, genetic expansion within Taiwan, and prehistoric settlements of some NAN_Tw groups in Taiwan predating the substantial demographic movements of individuals from Fujian and Guangdong in the last 200 years. To support this hypothesis, we reviewed Wang et al. (2021)^55^ analysis of complete human genome sequencing of nine ancient human remains (at ~1.5 kya) from the Hanben archaeological site in northeastern Taiwan^55^. Its most recent dating could be from the early iron age (1.5 to 0.4 kya). Among their genetic dataset, three ancient remains carried the Y haplogroup O3a2b*-M7, and six carried O3a2c*-P164 (now officially renamed O2a2a1a2-M7 and O2a2b-P164, respectively). These Y-chromosome haplogroups are scarce among Austronesian speakers of insular Asia and are mainly associated with NAN individuals from mainland Asia. In agreement with our observation (exclusive mtDNA haplogroups with an age range of 1.0 to 2.6 kya), these findings corroborate late Neolithic era to early metal age (1.6 kya) settlements of non-Austronesian-speaking individuals in Taiwan^54^. Our previous study on archaeological human remains of the Neolithic Ling-Ding site II near Hualien in Taiwan (Fig. 1)^56^ characterized four human remains with mtDNA haplogroups (B4b, C4a2, N9a1, and Z). Similarly, these haplogroups, commonly seen in modern urban Taiwanese and continental Asia, support the findings of our study and the possibility of early settlements in Taiwan of non-Austronesian speakers from continental Asia. The BAPS analyses (Fig. 3) arranged the studied groups into four clusters of shared ancestry: (a) a cluster comprising Japan, China groups, and NAN Taiwan groups principally represented by Ur_Tw and LL_Tw, (b) a Malaysian cluster receiving 15% gene flow from Indonesia and MSEA (Thailand and Vietnam), less than 3.4% from China groups and NAN_Tw, and 2% from AN_Tw and the Philippines, (c) an Austronesian cluster comprising AN_Tw, the Philippines, and Makatao groups showing gene flow (2%) toward NAN_Tw, (d) an Indonesia and MSEA cluster indicating a distant relationship (6%) with NAN_Tw.

The northward gene flow from ISEA/MSEA toward Taiwan (6%) seen in Fig. 3 was also suggested by the MDS and DAPC analyses (Figs. 2 and 3) and is evidenced by the sharing of NAN haplogroups (B4a1, B4c1b2a, B4c2, B5a, F1a, F1a1a, M7c1, and M20) between Indonesia, MSEA, South China, and Fujian (Supplementary Table S1). Interestingly, haplogroups M7c1, R9, and M20 were also reported in India and Eurasia and imply ancient gene flows of NAN individuals throughout Southern and Eastern Asia, likely representing fingerprints of migrations and/or trading networks throughout the China Sea^12^. Although only indicating an undated gene flow through the China sea channel, this observation is in agreement with reports on archaeological sites suggesting trading networks of Chinese ceramic from the 12th century AD characterized in Penghu Island and the north and south of Taiwan^12^. The evidence of trading systems and cross-regional cultural exchanges between groups of Southern Asia, Eastern Asia, and Taiwan since 400 BC was further suggested by deposits containing glass beads and metal age materials of this period found in the archaeological sites of Jiuxianglan in Southeast Taiwan^54^. Complete mtDNA genome analysis of these haplogroups and their distribution through MSEA, East Asia, ISEA, and Taiwan and complete human genome analysis should help give more support to these observations.

## Conclusion

The substantial distribution of mtDNA diversity found among non-Austronesian speakers of Taiwan and their relationship with neighboring Asian groups offers a better understanding of the matrilineal structure of Taiwan. This is likely the result of repeated cultural influences from various non-Austronesian human settlements from mainland Asia over prehistoric and historic periods. To a lesser extent, it is also the result of Malayo–Polynesian interactions through trade between Western Indonesia and mainland Southeast Asia. Rapid progress in molecular genetics will reduce the stochastic effect from the analysis of uni-parental systems. However, this study contributes to a better genetic characterization of NAN-speaking Taiwanese individuals. It exposes the importance of using other gene systems and analyzing other ethnic groups on the island before pertinent information needed to unravel their genetic heritage becomes unreachable.

### Supplementary information

Haplogroup diversity and distribution

Phylogenetic tree of novel sub-haplogroups found among AN_Tw

Mismatch distribution and Bayesian skyline plots

Heat map of gene flow (p = 0.01) for K=30

Network of Haplogroups B4, F1, M8, N9, R9

Network of Haplogroups D4a, D5, M7b, and M7c1a

Ancestry Mixture (p = 0.01, n ≥ 20) K2-K30

Delta K

Haplogroup Frequency using this study and literature datasets

Extant Mixture with two Putative Parent Populations (Han and Austronesian-speaking groups)

Dataset 1

Supplementary Material

## Data Availability

We deposited the new whole-mtDNA sequences used in this study in NCBI GenBank under accession numbers OL505314-OL505398 and MT954925-MT954932.
