# Supplementary material for: Diversity and distribution of mitochondrial DNA in non-Austronesian-speaking Taiwanese individuals
Source: Hum Genome Var. 2023 Jan 18;10:2. doi: 10.1038/s41439-022-00228-3 (PMC9849472; doi:10.1038/s41439-022-00228-3)
Supplement: Supplementary file 1 — Haplogroup diversity and distribution [file 41439_2022_228_MOESM1_ESM.docx]

**Title: Diversity and Distribution of Mitochondrial DNA Haplogroups in Non-Austronesian Speaking Taiwanese**

Marie Lin^1*^, Jean A. Trejaut^1*^

^1^Molecular Anthropology and Transfusion Medicine Research Laboratory, Mackay Memorial Hospital, Taipei, Taiwan

**Authors' email addresses:**Lin, Marie marielin0530@gmail.com
Trejaut, Jean Alain jeantrejaut@yahoo.com

^*^**Address for corresponding authors:**
Email: marielin0530@gmail.com or [jeantrejaut@yahoo.com](mailto:jeantrejaut@yahoo.com)

**Supplementary Information**

**Haplogroup diversity and distribution**

1. **Distribution of novel haplogroups of the N branch (complete mtDNA genome)**

*Haplogroup N (at ~51.6 kya).*
Haplogroups A, B, F, R, and Y are branches of macro-haplogroup N. They prevail in East, Southeast, Central, and Mainland Southeast Asia ^1^. Haplogroup N9 has raised particular interest for its association with Type 2 Diabetes in Japan but not in Taiwan ^2,3^. In Taiwan, N9a (~3.3%) is mostly seen among LL_Tw and Ur_Tw. We found six new lineages forming three sub-branches of haplogroup N9a (Supplementary Figures S1 and S4):
_Three sister lineages are forming the N9a3_10231 sub-clade (at ~2.6 kya). This haplogroup is scarcely seen in LL_Tw and Fujian.
_Two LL_Tw individuals bear the novel haplogroup N9a4b1_16291 seen throughout China at low frequency.
_Two sister twigs of N9a9, here named N9a9_16390 are seen throughout Continental and insular East Asia.
Interestingly, haplogroup N9a10a seen at low frequency among LL_Tw (0.08%) is likely the result of a mixture of Lowlanders with AN_Tw groups where it is more commonly seen among the Atayal indigenous group (~6%) ^4^ (Supplementary Table S1).

*Haplogroup A.*
Haplogroup A (~50 kya) is an early branch of macro-haplogroup N. It is seen in North America and shows its greatest variety in East Asia ^5^. In this study, members of haplogroup A are seen as sub-haplogroup of A5 (Supplementary Figure S1) with a TMRCA of approximately 10 kya BP (Table 2), which prevails in populations of Korea and Japan. Haplogroup A5b1c likely reached South China (~1.5%) from Northeast China before reaching Taiwan (Supplementary Table S1). Similarly, haplogroup A20 defined by nps 16362 and 14696 is usually found in North East China and Japan. The lineage described here showed an exclusive nucleotide position at 16148 (at ~2.7 kya) and was seen at least once in most Taiwan ethnic groups (Supplementary Figure S5).

*Haplogroup B.*

Haplogroups B4a, B4b, and B4c (Supplementary Figure S1 and S4) prevail in North and East Asia, and are seen in AN and NAN Taiwanese speakers. Haplogroups B5a1 and B5b1, less common in Taiwan are more frequently seen in MSEA and the Philippines ^6,7^. Other sub-types of B are scarcely seen in Taiwan. With a coalescence of 18.8 kya, the B4 clade is the most polymorphic. We characterized eleven new B sequence lineages, eight were direct branches of the B4 node, and one lineage in each of the B4h, B5 and B6 clades (Supplementary figure S1). Overall, five lineages were exclusive to NAN_Tw (B4c1b2b_16305, B4c1b2c1_16280, B4c1b2c2_10250, B4a_16234 and B5a_16260/16274). Haplogroups B4b1a2a_16309 and B4c1b2a_16242 were seen throughout Fujian and MSEA, and B4a5_8730 was seen in Fujian. In general, the NAN_Tw B diversity shows more sharing with Continental East and Southeast Asia.

*Haplogroup R.*

Haplogroup R (Supplementary Figure S1 and S4) has a wide diversity in the indigenous populations of South and East Asia ^8^. However, in East Asia and Taiwan its diversity is mostly restricted to sub-clades of R9b1 (at ~16.2 kya) and R9c (at ~6.7 kya). R9b appears in the Philippines, Indonesia, Aboriginal Malays, and is seen at low frequency among NAN_Tw (Supplementary Table S1). Similarly, haplogroup R9c is seen in West Indonesian, the Philippines and among Negritos of Palawan, and prevails among Ur_Tw ^9–12^. We defined five new sequence lineages, one defined as haplogroups R9b1a2b_16319 (at~11.5 kya), two as haplogroup R9b1a3_16319 (at~3.9.5 kya), and two twigs from novel haplogroup R9c_10403 (at~6.3kya). These haplogroups suggest a of ancestry with the Philippines, and to a various degree, South China and West Indonesia.

*Haplogroup F.*Haplogroup F has a coalescence of 45.7 kya and prevails throughout Continental and Island Southeast Asia. The major sub-haplogroups seen in LL_Tw are F1a (at ~26.8 kya, and 7.95%), F2 (at 22.9 kya and 3.72%), F3 (1.41%), and F4 (at 41.5 kya and 0.8%) (Supplementary Table S1). Most F subtypes in NAN_Tw were also seen in AN_Tw, Fujian, ISEA, and MSEA (Vietnam and Thailand). The identification of thirteen novel F sequence lineages allowed a better characterization of the F1a1 clade. Except for F1a1_8265, seen in Taiwan and Japan (Supplementary Figure S1 and S4), the other sub-clades of F1a1 (F1a1_10463; at ~8.9 kya, and F1a1a_16311; at ~1.3 kya) were also seen in MSEA and West Indonesia. Lastly, four lineages with coalescence time ranging from 2.8 to 4.6 kya were exclusive to NAN_tw (F2_8264, F2_10313, F4a2_16243, and F4b1_8665) (Supplementary Figure S1 and S4).

1. **Distribution of novel haplogroups of the M branch (complete mtDNA genome)**

*Haplogroup M7.*
Haplogroup M7 is seen throughout East Asia and has an approximate TMRCA of 45.6 kya (Table 2). Most lineages of M7 seen among NAN_Tw belonged to twigs of two major clades, M7b1a1 (6.28%) and M7c1 (4.1%) (Supplementary Table S1, Figure S1, and S5). These clades display strong Southeast Asia and MSEA ancestry.
*The M7b1a clade.*With a coalescence estimate of ~13 kya and a polymorphism shared with South China and MSEA, the M7b1a clade suggests a pre-Holocene expansion external to Taiwan. This clade also comprises five novel lineages (Supplementary Figure S1 and S5) of which M7b1a1a1b_16162 and M7b2a_8389 are exclusive to NAN_Tw (Supplementary Table S1). The TMRCA (Table 2) of these novel lineages (at ~2.6 kya) suggest post-AN agriculturist migrations with likely pre-historical expansion restricted to Taiwan ^13^.

The M7c1a clade

The M7c1a clade is seen throughout East Asia, and among LL_Tw (1.54%). This clade was characterized as M7c3c2 with Haplogrep 2, however, complete genome sequencing characterized it as M7c1a3 with nps 9824, 16295 and 16319. A back mutation at np 16223 (i.e. C16223T) (Supplementary Figure S1 and S5) characterized six novel twigs of haplogroup M7c1a3a: M7c1a3_1664 ^14^ and five novel lineages forming sub-clades M7c1a3_469 and M7c1a3_3027. Members of these clades finds their origin in Fujian/Southeastern China five to seven kya (Table 2). Other minor sub-clades of M7c1a (M7c1a1a, M7c1a3, M7c1a4b, and M7c1a5) are seen at very low frequencies in Taiwan. Ten lineages, specific to LL_Tw, were determined on the tips of these clades. They dated approximately 2.6 kya, suggesting they likely arrived along with bearers of M7c1a4, from South China or East China (Fujian) in the late Neolithic.

Last, the scarce presence in NAN_Tw of M7c1c3 along with B4c1c, F1a4a, and Y2, have been proposed as markers for the “out-of-Taiwan” dispersal ^13^. Their scarcity among NAN_Tw indicates there was little contact between LL_Tw of Taiwan’s west coast, and Taiwan’s Indigenous peoples on the East coast.

*Haplogroup M8.*

Haplogroup M8 prevails in East Siberia, North China, and Korea. Its sub-clade M8a2 (at ~15.5 kya) spread widely in the late Paleolithic throughout China, MSEA, and reached a frequency of 14% in China East Coast (Fujian) (Supplementary Table S1) ^13^. Haplogroup M8a2 is locally defined by three nucleotide variations at nps 16470, 16471, and 16473. Its sub-types show significant diversity among NAN_Tw (Supplementary Figure S1). Three novel haplogroups were defined: haplogroups M8a2a1_8410, M8a2a1_8245, and M8a2_8503 (Supplementary Figure S4). Haplogroup M8a2a1 (1.87%) finds its origin in South China, along with branches in MSEA and Fujian ^15^. Haplogroups M8a2_8503 and M8a2a1_8410 were exclusive to NAN_Tw, an average coalescence estimate of less than 1.7 kya suggests M8a2_8503 and M8a2a1_8410 may have gained their diversity locally in Taiwan.

*Haplogroup C and Z*.
Haplogroup C and Z are branches off the M8 clade. Our protocol differentiates haplogroups M8a, C, and Z with nps 8584, 16327, and 16260, respectively. Haplogroup C is widespread across East Asia and MSEA ^12,16^. A novel C lineage in the C7a clade (C7a_10310; 0.28%) (Supplementary Figure S1) was found in Ur_Tw. Its scarcity and a coalescence time estimate of 12 kya infer past expansion in MSEA and a recent migration to Taiwan.
Haplogroup Z4 prevails in Southern China. It has also been reported in Northeast China, Japan, the Kazakhs, the Himalaya, and Northern Thailand. It was not seen among AN_Tw. Our screening method defined it with np 16260 and np 16248, however, the complete genome sequencing lacked np 15944 usually used to determine Z4 ^17^. Accordingly, we classified it as a novel lineage (haplogroup Z4_16248) that is exclusively seen among NAN_Tw. Its TMRCA at ~1.3 kya (Table 2) suggests a late Neolithic migration of NAN speakers from the mainland to Taiwan.

*Haplogroup D.*

D4 is most frequently seen in northern East Asia. In this study D4 also prevails among NAN_Tw (9.74%). We found novel lineages within the D4a3b ^3^ clade (Supplementary Figure S1 and Figure S5) tentatively named D4a3b_16274 (also characterized with nps 7805 and 9738), and D4a3b2_16278 (also characterized with nps 3759 and 11140). Further, D4a3b2 (at ~1.6 kya) defined with nps 12892 and 16278, revealed a novel lineage characterized by nps 3759 and 11140.
In the D4b1 clade, D4b1b2_16380 was not charted in Phylotree 17, however its sister lineages were previously described ^18,19^ among Northeastern China and Japan (Supplementary Figure S1 and Figure S5).
The D4b2 clade is primarily found at low frequency in Eastern China ^20,21^ and is scarcely seen in Taiwan. However, two exclusive novel NAN_Tw lineages were determined: D4b2b_10310, and D4b2b2b_16274 (Supplementary Figure S1).
Last, the D5 clade (Supplementary Figure S1 and Figure S5) is mostly found throughout East China, Korea and Japan. With a frequency of 7% in Taiwan, the polymorphism of D5a2 and D5b1 are well represented. One novel lineage in each of these clades was found to be specific to NAN_Tw (D5a2a1b1_16293 and D5b1b2_16249).

**References**

1. Palanichamy, M. G. *et al.* Phylogeny of mitochondrial DNA macrohaplogroup N in India, based on complete sequencing: implications for the peopling of South Asia. *Am J Hum Genet* **75**, 966–978 (2004).

2. Fang, H. *et al.* mtDNA haplogroup N9a increases the risk of type 2 diabetes by altering mitochondrial function and intracellular mitochondrial signals. *Diabetes* **67**, 1441–1453 (2018).

3. Loo, J. H. *et al.* Mitochondrial DNA association study of type 2 diabetes with or without ischemic stroke in Taiwan. *BMC Res Notes* **7**, 223 (2014).

4. Ko, A. M. *et al.* Early Austronesians: into and out of Taiwan. *Am J Hum Genet* **94**, 426–436 (2014).

5. Fagundes, N. J., Kanitz, R. &Bonatto, S. L. A reevaluation of the Native American mtDNA genome diversity and its bearing on the models of early colonization of Beringia. *PLoS One* **3**, e3157 (2008).

6. Derenko, M. *et al.* Phylogeographic analysis of mitochondrial DNA in northern Asian populations. *Am J Hum Genet* **81**, 1025–1041 (2007).

7. Duong, N. T. *et al.* Complete human mtDNA genome sequences from Vietnam and the phylogeography of Mainland Southeast Asia. *Sci Rep* **8**, 11651 (2018).

8. Larruga, J. M., Marrero, P., Abu-Amero, K. K., Golubenko, M.V. &Cabrera, V. M. Carriers of mitochondrial DNA macrohaplogroup R colonized Eurasia and Australasia from a southeast Asia core area. *BMC Evol. Biol.* **17**, (2017).

9. Hill, C. *et al.* Phylogeography and ethnogenesis of aboriginal southeast asians. *Mol Biol Evol* **23**, 2480–2491 (2006).

10. Scholes, C. *et al.* Genetic diversity and evidence for population admixture in Batak Negritos from Palawan. *Am J Phys Anthr.* **146**, 62–72 (2011).

11. Delfin, F. *et al.* Complete mtDNA genomes of Filipino ethnolinguistic groups: a melting pot of recent and ancient lineages in the Asia-Pacific region. *Eur J Hum Genet* **22**, 228–237 (2014).

12. Kutanan, W., Kampuansai, J., Brunelli, A. &al., et. New insights from Thailand into the maternal genetic history of Mainland Southeast Asia. *Eur J Hum Genet* 898–911. https://doi.org/10.1038/s41431-018-0113–7 (2018).

13. Soares, P. A. *et al.* Resolving the ancestry of Austronesian-speaking populations. *Hum Genet* **135**, 309–326 (2016).

14. Chen, Z.-S. *et al.* Mitochondrial DNA Diversity of the Nangan Islanders Living in the Mazu Archipelago of the Taiwan Strait. *Edelweiss J. Biomed. Res. Rev.* **3**, 25–27 (2021).

15. Luo, X. Q. *et al.* Uniparental Genetic Analyses Reveal the Major Origin of Fujian Tanka from Ancient Indigenous Daic Populations. *Hum Biol* **91**, 257–277 (2020).

16. Fairley, S., Lowy-Gallego, E., Perry, E. &Flicek, P. The International Genome Sample Resource (IGSR) collection of open human genomic variation resources. *Nucleic Acids Res.* **48**, D941–D947, https://doi.org/10.1093/nar/gkz836 (2020).

17. vanOven, M. &Kayser, M. Updated comprehensive phylogenetic tree of global human mitochondrial DNA variation. *Hum Mutat* **30**, E386-94 (2009).

18. Kong, Q.-P. *et al.* Phylogeny of East Asian mitochondrial DNA lineages inferred from complete sequences. *Am J Hum Genet* **73.**, 671–676 (2003).

19. Tanaka, M. *et al.* Mitochondrial genome variation in eastern Asia and the peopling of Japan. *Genome Res* **14**, 1832–1850 (2004).

20. Zhao, D. *et al.* Mitochondrial Haplogroups N9 and G Are Associated with Metabolic Syndrome Among Human Immunodeficiency Virus-Infected Patients in China. *https://home.liebertpub.com/aid* **35**, 536–543 (2019).

21. Kong, Q. P. *et al.* Large-scale mtDNA screening reveals a surprising matrilineal complexity in east Asia and its implications to the peopling of the region. *Mol Biol Evol* **28**, 513–522 (2011).

22. Bandelt, H. J., Forster, P. &Rohl, A. Median-joining networks for inferring intraspecific phylogenies. *Mol Biol Evol* **16**, 37–48 (1999).

23. Barrett, J. C., Fry, B., Maller, J. &Daly, M. J. Haploview: analysis and visualization of LD and haplotype maps. *Bioinformatics* **21**, 263–265 (2005).
