# Supplementary figures and images for: Diversity and distribution of mitochondrial DNA in non-Austronesian-speaking Taiwanese individuals
Source: Hum Genome Var. 2023 Jan 18;10:2. doi: 10.1038/s41439-022-00228-3 (PMC9849472; doi:10.1038/s41439-022-00228-3)

# Supplementary Figure S1

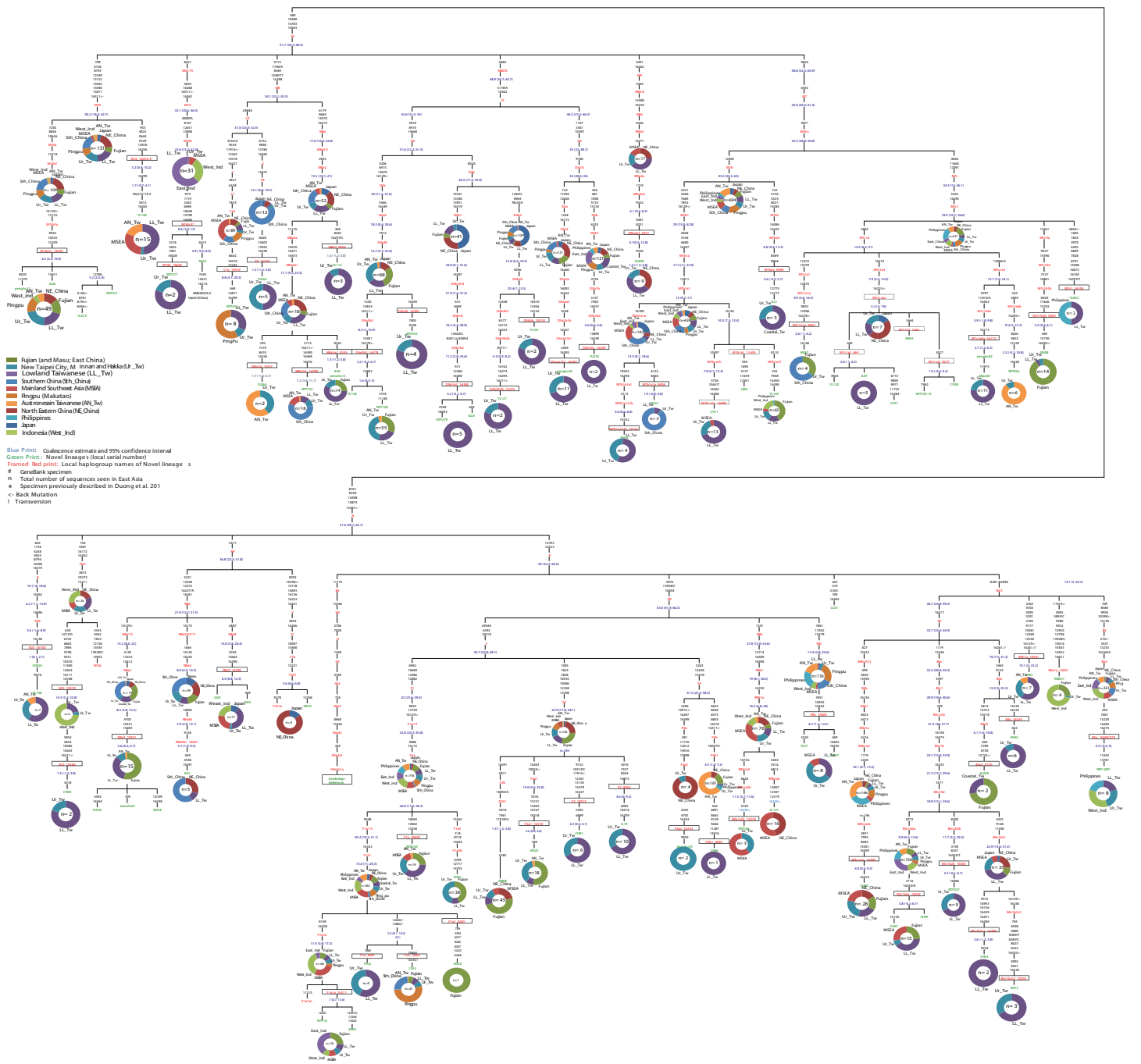

Supplement: Supplementary file 2 — Phylogenetic tree of novel sub-haplogroups found among AN_Tw [file 41439_2022_228_MOESM2_ESM.pdf]

Supplementary Figure S3: Gene flow matrix (Rows = source, and Column = target; P value:0.01;K=30)

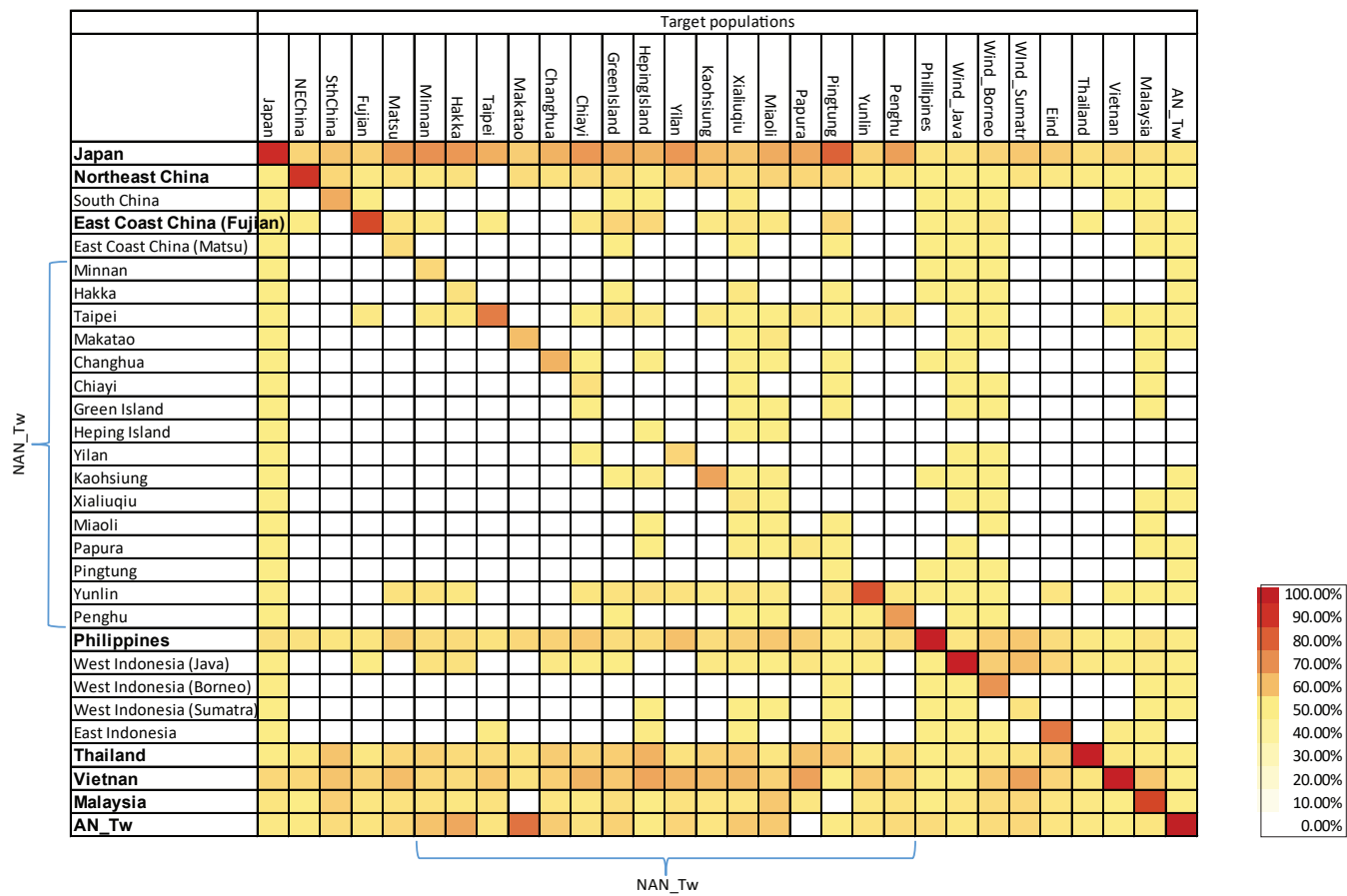

Supplement: Supplementary file 4 — Heat map of gene flow (p = 0.01) for K=30 [file 41439_2022_228_MOESM4_ESM.pdf]

Supplementary Figure S6. Ancestry Mixture (n>20: K2-K30)

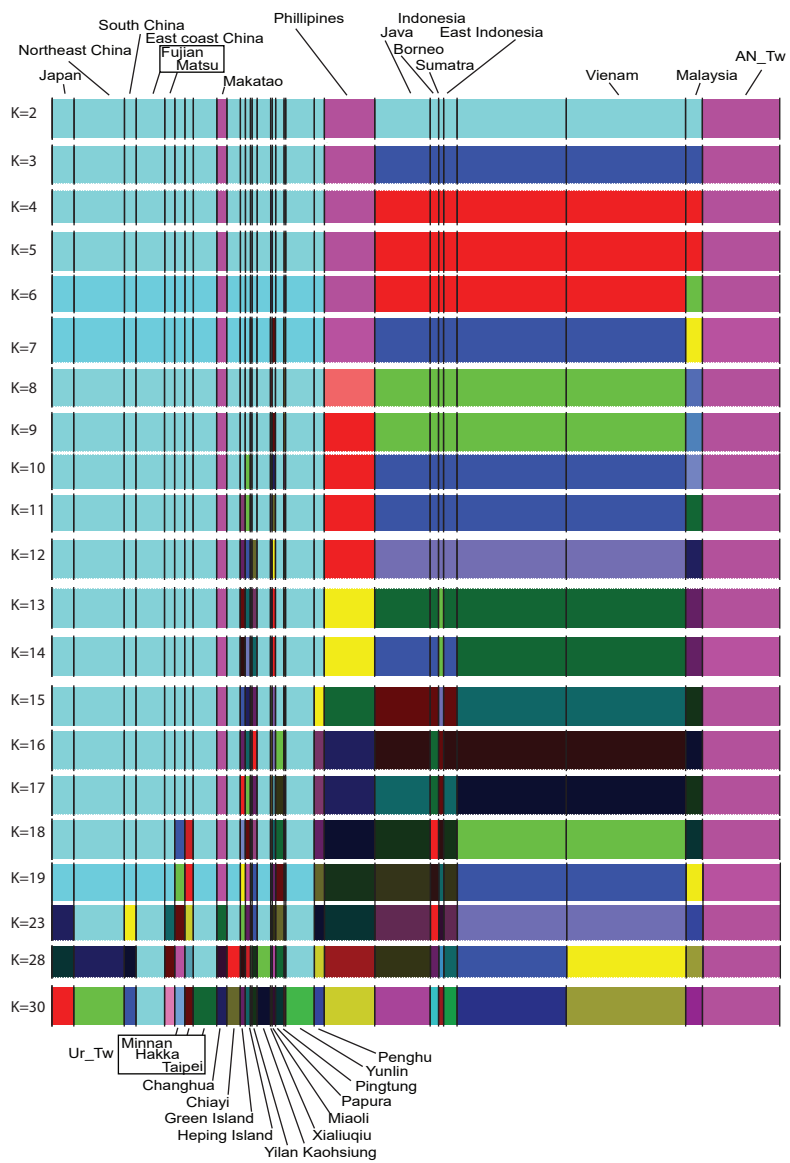

Supplement: Supplementary file 7 — Ancestry Mixture (p = 0.01, n ≥ 20) K2-K30 [file 41439_2022_228_MOESM7_ESM.pdf]
