# Supplementary material for: Diversity and distribution of mitochondrial DNA in non-Austronesian-speaking Taiwanese individuals
Source: Hum Genome Var. 2023 Jan 18;10:2. doi: 10.1038/s41439-022-00228-3 (PMC9849472; doi:10.1038/s41439-022-00228-3)
Supplement: Supplementary file 3 — Mismatch distribution and Bayesian skyline plots [file 41439_2022_228_MOESM3_ESM.pdf]

Supplementary Figure S2. Mismatch distribution and Bayesian skyline plots

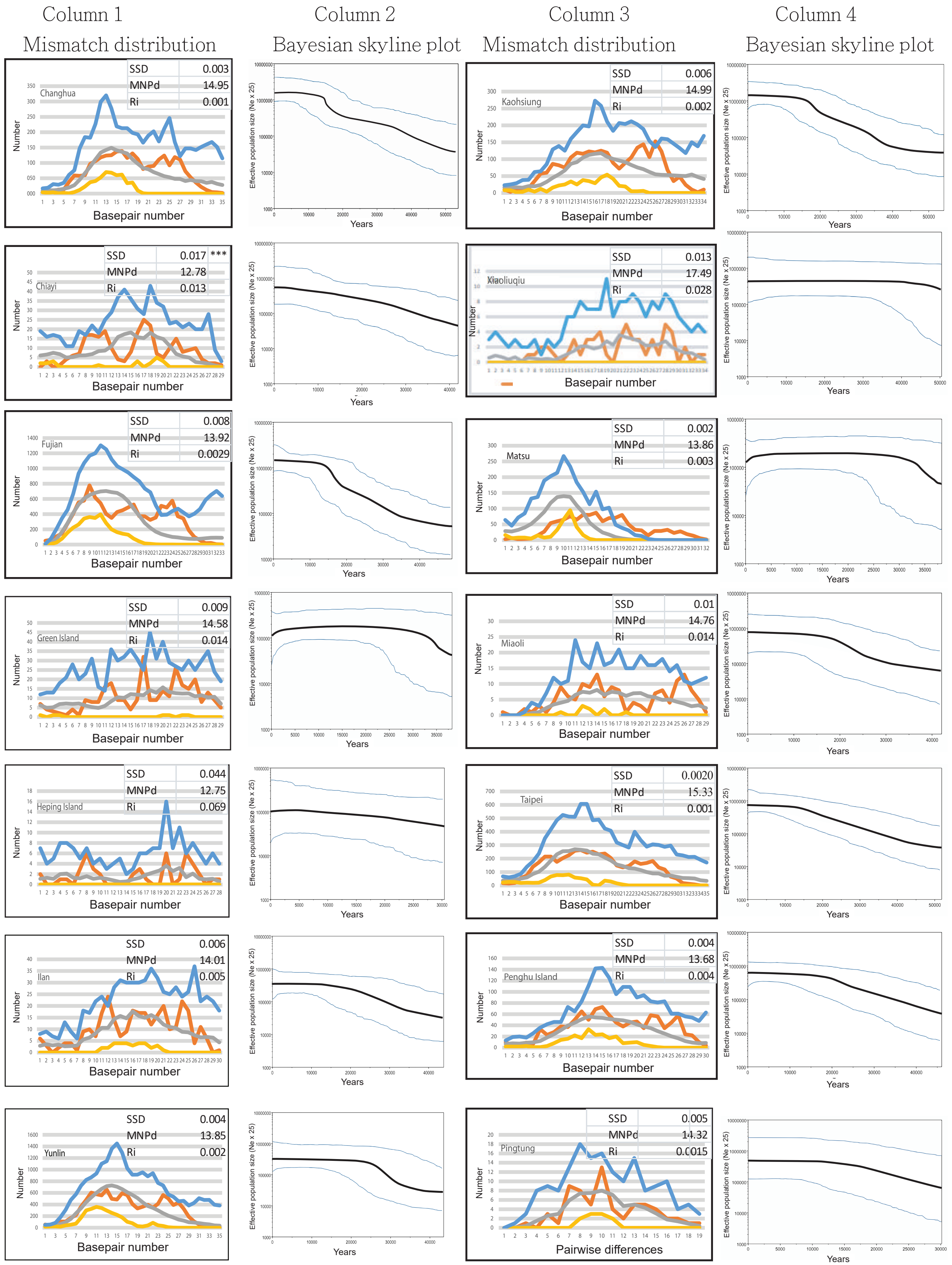

Observed Mismatches: Lowbound:
Simulated Mismatches: Upbound :

*: p ≤ 0.05; **: p ≤ 0.01; ***: p ≤ 0.001; Ri: Ragedness Index; SSD: Sum of square difference; MNPd: Mean number of pairwise differences
