# Supplementary material for: Diversity and distribution of mitochondrial DNA in non-Austronesian-speaking Taiwanese individuals
Source: Hum Genome Var. 2023 Jan 18;10:2. doi: 10.1038/s41439-022-00228-3 (PMC9849472; doi:10.1038/s41439-022-00228-3)
Supplement: Supplementary file 6 — Network of Haplogroups D4a, D5, M7b, and M7c1a [file 41439_2022_228_MOESM6_ESM.pdf]

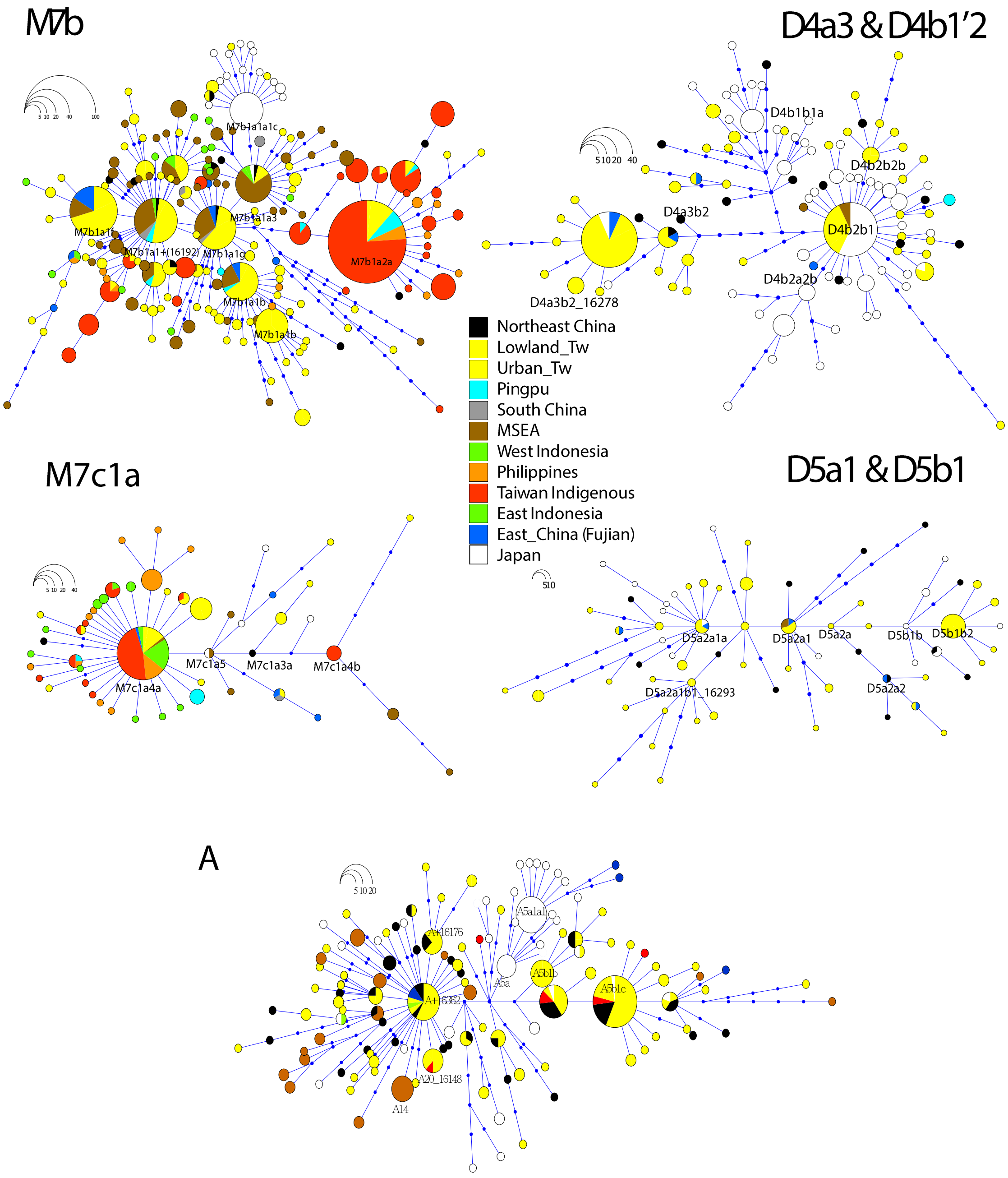

Supplementary Figure S5. Network of Haplogroups D4a, D5, M7b, and M7c1a Networks for the concatenated regions of HVS-I at nps 16051 to 16400, and coding regions at nps 8001 to 9000 and 9801 to 10900 were first generated with the minimum-reduced algorithm, then using the median-joining algorithm for estimating coalescence time using one site per 8,940 years. Graphic plots were processed with the Haploview package version 4.2[1].

Reference

1. Barrett, J. C., Fry, B., Maller, J. &Daly, M. J. Haploview: analysis and visualization of LD and haplotype maps. *Bioinformatics* **21**, 263–265 (2005).
