# Supplementary material for: Diversity and distribution of mitochondrial DNA in non-Austronesian-speaking Taiwanese individuals
Source: Hum Genome Var. 2023 Jan 18;10:2. doi: 10.1038/s41439-022-00228-3 (PMC9849472; doi:10.1038/s41439-022-00228-3)
Supplement: Supplementary file 8 — Delta K [file 41439_2022_228_MOESM8_ESM.pdf]

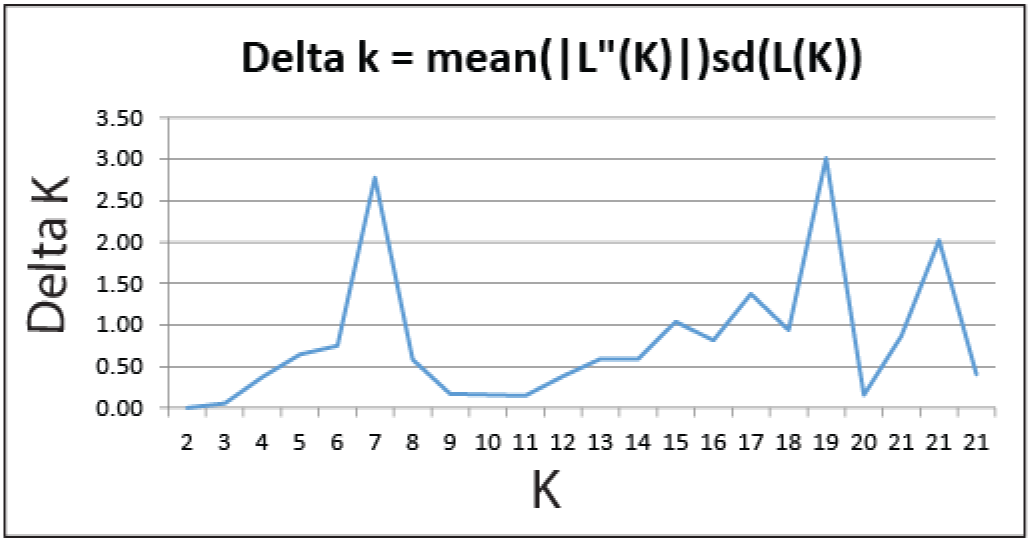

Supplementary Figure S7. Delta K
Determination of the uppermost hierarchical level of structure (Delta K) using the Evanno statistic[1]. Mixture analysis with K = 2 to 22 was carried out to determine the Delta K.

Reference

1. Evanno, G., Regnaut, S. & Goudet, J. Detecting the number of clusters of individuals using the software structure: a simulation study. *Mol. Ecol.* **14**, 2611–2620 (2005).
