# Supplementary material for: Diversity and distribution of mitochondrial DNA in non-Austronesian-speaking Taiwanese individuals
Source: Hum Genome Var. 2023 Jan 18;10:2. doi: 10.1038/s41439-022-00228-3 (PMC9849472; doi:10.1038/s41439-022-00228-3)
Supplement: Supplementary file 12 — Supplementary Material [file 41439_2022_228_MOESM12_ESM.docx]

**Supplementary Material**

**Supplementary Text S1.** Haplogroup diversity and distribution

**Supplementary Tables**

**Supplementary Table S1**. Haplogroup frequency using this study and literature datasets

**Supplementary Table S2**. Extant Mixture with two Putative Parent Populations (Han and Austronesian speaking groups)

**Supplementary Table S3.** Mitochondrial DNA sequences from 672 coastal Taiwanese individuals analyzed using HVS-I and coding regions 8001-9000 and 9001-10900

**Supplementary Figures**

**Supplementary Figure S1**. Phylogenetic tree of novel subhaplogroups found among AN_Tw. Colors used in Pie charts indicate the relative geographic distribution as described in the insert. The numbers within the Pie charts and coalescence times were obtained from a dataset of the complete mtDNA genome obtained from this study, the literature dataset (Table 1), and Phylotree 17. The TMRCA of haplogroups is provided only when more than three lineages are available for the clade.

**Supplementary Figure S2**. Mismatch distribution and Bayesian Skyline plots.
Columns 1 and 3: Mismatch distribution plots, where the y-axis is the number of observed individuals and the x-axis is the number of shared base pairs. Fujian and Matsu do not support the model of sudden expansion.
Columns 2 and 4: Bayesian Skyline plots.
The y-axis is the log scale (product of effective population size (Ne) and generation time (t=25 years per generation); the x-axis is the time in thousand years BP. The central line is the mean; dashed upper and lower lines are the 95% highest posterior density interval (HPD).
By 10,000 BP, continental and urban groups show effective population sizes (N_e_) greater than 18,000. Comparatively, most LL_Tw N_e_ range to approximately 3500 individuals, and islander groups such as Green Island and Matsu show a low N_e_ with a decrease in the last few thousand years.

**Supplementary Figure S3**. Heatmap of gene flow (p = 0.01) for K=30.
The network of population clusters with a relative average of ancestry/gene flow was constructed using K=30 and a significance threshold of p = 0.01. Only group sizes ≥ 20 were considered. The cell colors represent the distribution of gene flow values from the lowest levels of exchange 0 (white) to the highest 1 (dark brown). Details of the populations are given in Supplementary Table S1. Rows represent source populations, and columns represent target populations. Note: The Ancestry Mixture plot K2 to K30 is also shown in Supplementary Figure S6.

**Supplementary Figure S4**. Network of Haplogroups B4, F1, M8, N9, R9
Networks for the concatenated regions of HVS-I at nps 16051 to 16400, and coding regions at nps 8001 to 9000 and 9801 to 10900 were first generated with the minimum reduced algorithm, then using the median-joining algorithm for estimating coalescence time using one site per 8,940 years. Graphic plots were processed with Haploview package version 4.2 ^1^.

**Supplementary Figure S5**. Network of Haplogroups D4a, D5, M7b, and M7c1a
Networks for the concatenated regions of HVS-I at nps 16051 to 16400, and coding regions at nps 8001 to 9000 and 9801 to 10900 were first generated with the minimum-reduced algorithm, then using the median-joining algorithm for estimating coalescence time using one site per 8,940 years. Graphic plots were processed with the Haploview package version 4.2 ^1^.

**Supplementary Figure S6**. Ancestry Mixture (p = 0.01, n ≥ 20) K2-K30
Results of genetic assignment of individual analysis are based on the Bayesian method implemented in the BAPS program under a mixture model for K = 2 to 30.

**Supplementary Figure S7.** Delta K

Determination of the uppermost hierarchical level of structure (Delta K) using the Evanno statistic ^1^. Mixture analysis with K = 2 to 22 was carried out to determine the Delta K.
